# Dysregulated Gene Expression in Lymphoblasts from Parkinson’s Disease

**DOI:** 10.3390/proteomes10020020

**Published:** 2022-06-01

**Authors:** Sarah Jane Annesley, Claire Yvonne Allan, Oana Sanislav, Andrew Evans, Paul Robert Fisher

**Affiliations:** 1 Department of Microbiology, Anatomy, Physiology and Pharmacology, La Trobe University, Melbourne, VIC 3086, Australia; claire.allan@latrobe.edu.au (C.Y.A.); o.sanislav@latrobe.edu.au (O.S.); p.fisher@latrobe.edu.au (P.R.F.); 2 Department of Neurology, Royal Melbourne Hospital, Parkville, VIC 3052, Australia; evaa@unimelb.edu.au

**Keywords:** Parkinson’s disease, proteome, transcriptome, protein synthesis, mitochondria, oxidative phosphorylation, cell models

## Abstract

Parkinson’s disease is the second largest neurodegenerative disease worldwide and is caused by a combination of genetics and environment. It is characterized by the death of neurons in the substantia nigra of the brain but is not solely a disease of the brain, as it affects multiple tissues and organs. Studying Parkinson’s disease in accessible tissues such as skin and blood has increased our understanding of the disease’s pathogenesis. Here, we used lymphoblast cell lines generated from Parkinson’s disease patient and healthy age- and sex-matched control groups and obtained their whole-cell transcriptomes and proteomes. Our analysis revealed, in both the transcriptomes and the proteomes of PD cells, a global downregulation of genes involved in protein synthesis, as well as the upregulation of immune processes and sphingolipid metabolism. In contrast, we discovered an uncoupling of mRNA and protein expression in processes associated with mitochondrial respiration in the form of a general downregulation in associated transcripts and an upregulation in proteins. Complex V was different to the other oxidative phosphorylation complexes in that the levels of its associated transcripts were also lower, but the levels of their encoded polypeptides were not elevated. This may suggest that further layers of regulation specific to Complex V are in play.

## 1. Introduction

Parkinson’s Disease (PD) is the second most common neurodegenerative disease worldwide and, collectively, neurodegenerative diseases are set to eclipse cancer as the leading cause of death by 2040 [1]. Diagnosis is based on clinical appearance of four cardinal features: bradykinesia, resting tremor, rigidity, and postural and gait impairment, as was first described by James Parkinson in 1817 [2].

PD is a complex disease generally classified as a sporadic or idiopathic disorder and caused by a combination of genetics and environment. Rarely, PD is caused by a monogenic mutation (10%). There are also several genes which have been identified as risk factors for PD; these do not usually result in loss or gain of function but rather a change in expression levels [3]. The genes associated with monogenic forms of PD or risk factors have diverse functions and do not explain the underlying causal mechanisms [3]. In all cases though, there are shared defects in common pathways including, but not limited to, mitochondrial function, protein aggregation, vesicle trafficking and autophagy, and inflammation and immune pathways. Dissecting these altered pathways and determining when their function is impaired in the progression of the disease will aid in the development of therapeutic options and also in the identification of the causal mechanism(s) of the disease.

While PD is characterised by the death of neurons in the substantia nigra pars compacta area of the brain, it is becomingly increasingly apparent that PD is not simply a brain disease but that it is systemic, affecting multiple tissues and organs. Several theories exist as to why the neurons in the substantia nigra are more susceptible to cell death, which have been discussed in detail elsewhere [4,5]. As brain tissue can only be accessed post-mortem, the systemic nature of the disease means that more easily accessible tissue could be useful for the identification of biomarkers and altered pathways. One of the most accessible tissues in the human body is the blood. Blood has been postulated to reflect the mechanisms of PD pathogenesis that may occur at the central level. For example, the degradation of proteins through autophagy is reduced [6] and an increase in proteostasis chaperones is evident [7] in PBMCs from PD.

Given the multifactorial nature and complexity of PD, high-throughput analysis of gene transcripts via transcriptomics and of proteins via proteomics both offer valuable approaches for identifying thousands of genes and proteins that are differentially expressed in PD. Transcriptomic and proteomic studies have been performed in a range of PD cell types including brain tissue, cerebrospinal fluid (CSF), fibroblasts, serum, and peripheral blood mononuclear cells (PBMCs; reviewed in [8,9]). While there is a lack of consistency in the individual proteins identified in these studies, these techniques may be more useful for identifying altered pathways, novel mechanisms underlying PD, and evaluations of treatment efficacy.

There are some limitations in transcriptomic and proteomic studies of certain cell types. For example, RNA molecules are not stable and may be degraded in post-mortem tissue—affecting their ability to be sequenced [10]—and some samples contain multiple cell types in varying proportions, such as PBMCs, with different cell types having different expression profiles [11]. An alternative to the isolation of particular blood cell types is the use of immortalised lymphocytes, termed lymphoblastoid cell lines or LCLs. LCLs have been used successfully to investigate gene expression changes in Alzheimer’s Disease (AD) [12]. In AD, LCL transcriptomic studies have identified the altered expression of the lipoprotein receptor relative with 11 binding repeats (LR11) gene, and subsequent studies confirmed the reduction of the expression of this gene in AD brains [12].

Previously, we created LCLs from idiopathic PD (iPD) patients and discovered that these cell lines had elevated mitochondrial respiration, elevated reactive oxygen species, and elevated ATP steady state levels [13]. This was in contrast to the dogma of mitochondrial dysfunction in PD [14]. Since then, there have been more reports by ourselves and others of elevated mitochondrial respiration in diverse cellular models of PD. These include such models as *Dictyostelium discoideum* genetic PD models [15], fibroblasts from iPD patients [16], and neuroblastoma cell lines exposed to oligomeric α-synuclein fibrils [17]. The discrepancy between these results and previous reports of mitochondrial dysfunction may lie in the proliferative nature of these cells compared with the terminally differentiated or metabolically dormant cells in other studies such as post-mortem brain tissue or PBMCs. We previously postulated that the high mitochondrial respiration in proliferating cells or in *nigral* neurons may result in elevated reactive oxygen species (ROS), which would accumulate over time in cells that are not rapidly turned over (*nigral* neurons), resulting in mitochondrial damage and dysfunction [13].

Here, we analysed the whole cell proteomes and transcriptomes of iPD and healthy control LCLs and identified a large number of genes which were transcriptionally and translationally regulated. The regulation of some processes was in agreement at both the transcription and translation levels, e.g., gene products involved in protein biosynthesis were downregulated at both the transcriptional and translational level. In other biological processes, there were conflicting results between the two data sets. This was particularly isolated to the upregulated proteins and transcripts, and we found many more proteins which were upregulated than downregulated in the proteome. A major discordance was found in the expression of gene products involved in mitochondrial respiration; we found that whilst transcripts for mitochondrial oxidative phosphorylation (OXPHOS) complexes were downregulated in the transcriptome, complexes I–IV were in fact upregulated in the proteome, thereby suggesting that the elevated mitochondrial respiration observed in these cell lines is translationally regulated. Interestingly, despite an increase in mitochondrial respiration and an increase in the abundance of Complex I–IV proteins, Complex V subunits were, if anything, decreased in abundance—although the magnitude of this trend did not reach statistical significance. This suggests that the regulation of Complex V expression may involve different mechanisms than the other respiratory complexes, and that it is worthy of further exploration. Our data using LCLs support the notion that PD is a systemic disorder involving the dysregulation of cellular processes at many levels. This insight highlights the benefit of a multiomic approach.

## 2. Materials and Methods

### 2.1. Participant Cohort

The study was approved by Human Ethics Committee of La Trobe University and all participants were asked and gave informed consent for their involvement in this study. All participants belonged to one of two groups: idiopathic Parkinson’s disease patients (PD) or healthy controls. The Parkinson’s disease group were clinically diagnosed by movement disorder specialists (AE), while healthy controls were recruited separately and did not have any known neurological disorders or other conditions that might have been relevant to the study.

### 2.2. Transcriptomic Cohort

There were 20 healthy control participants: 14 male, 5 female, and 1 unknown, with an age range of 35–78 and a median age of 64. There were 20 PD participants: 19 males and 1 female, with an age range of 45–83 and a median age of 64—with age data missing for two PD participants. There was no difference in median age between the two groups and no significant difference in gender (Fisher’s exact test, *p*-value = 0.91).

### 2.3. Proteomic Cohort

In addition to the participants listed above for the transcriptomic cohort, the proteomic cohort consisted of an additional 8 healthy controls and an additional 27 PD participants. The total cohort therefore included 28 healthy control participants: 22 male and 5 female (1 unknown), with an age range of 35–83 and a median age of 66.5. There were 47 PD participants: 38 male and 9 female, with an age range of 28–83 and median age of 68. Four PD participants had data missing for age. The age proportions were not significantly different between the two groups (Mann–Whitney U test, *p*-value = 0.755) or gender proportions (Fisher’s exact test, *p* value = 1).

### 2.4. Generation of Lymphoblastoid Cell Lines

Lymphoblastoid cell lines were created by EBV-mediated transformation of peripheral blood mononuclear (PBMC) cells, as described in [13]. The cell lines were grown and maintained in MEM alpha medium (Thermo-Fisher Scientific, Waltham, MA, USA) supplemented with 10% foetal bovine serum (FBS; (Thermo-Fisher Scientific, Waltham, MA, USA)) and 1% Pen-Strep (Thermo-Fisher Scientific, Waltham, MA, USA) at 37 °C with 5% CO_2_. Cell lines were passaged for a total number of passages of less than 20.

### 2.5. Whole-Cell Protein Extraction and Mass Spectrometry

For proteome analysis, 3 × 10^6^ lymphoblastoid cell lines were harvested by centrifugation at 500× *g* for 5 min and resuspended in 100 µL of PBS (Sigma-Aldrich, St. Louis, MI, USA). The samples were then sent to the La Trobe University Comprehensive Proteomics Platform and processed according to the following protocol:

Each sample was dried using a SpeedVac Concentrator and Savant Refrigerated Vapor trap (Thermo-Fisher Scientific, Waltham, MA, USA). Samples were resuspended in 8 M urea (Sigma-Aldrich, St. Louis, MI, USA), 100 mM tris pH = 8.3 (Sigma-Aldrich, St. Louis, MI, USA). A volume of 1 µL of tris (2-carboxyethyl) phosphine hydrochloride (Sigma-Aldrich, St. Louis, MI, USA), (TCEP, 200 mM solution in water) was then added to the samples and incubated overnight at 21 °C in a ThermoMixer (Eppendorf AG, Hamburg, Germany). Four microliters of 1 M iodoacetamide (Sigma-Aldrich, St. Louis, MI, USA), (IAA in water) was added the following day and incubated in the dark at 21 °C. Next, 500 µL of 50 mM Tris (pH 8.3) and 1 µg trypsin was added to samples and left for 6 h at 37 °C in an incubator. Another 1 µg trypsin was added for double digestion and incubated overnight at 37 °C. The digested samples were purified for mass spectrometry analysis prior to peptide reconstitution and separation using Sep-Pak light C18 cartridges (Waters, Milford, CT, USA), according to manufacturer standard procedures. Data were collected on a Q Exactive HF (Thermo-Fisher Scientific, Waltham, MA, USA) in the Data-Dependent Acquisition mode using *m*/*z* 350–1500 as the mass spectrometry (MS) scan range at 60,000 resolution, HCD MS/MS spectra were collected for the 15 most intense ions per MS scan at 15,000 resolution, with a normalised collision energy of 28% and an isolation window of 1.4 *m*/*z*. Dynamic exclusion parameters were set as follows: exclude isotope on, duration 30 s, and peptide match preferred. Other instrument parameters for the Orbitrap were MS maximum injection time of 30 ms with an AGC target of 3 × 10^6^. Raw files consisting of high-resolution MS/MS spectra were processed with MaxQuant v. 1.6.1.0 (https://www.maxquant.org/, accessed on 19 May 2022) to detect features and identify proteins using the search engine Andromeda. UniProtKB/Swiss-Prot *Homo sapiens* (https://www.expasy.org/resources/uniprotkb-swiss-prot, accessed on 19 May 2022) sequence data were used as the database for the search engine. To assess the false discovery rate (FDR), a decoy dataset was generated by MaxQuant after reversing the sequence database. Up to two missed cleavages were permitted. The minimum required peptide length used was 7 amino acids. Carbamidomethylation of Cys was set as a fixed modification, while *N*-acetylation of proteins and oxidation of Met were set as variable modifications. Precursor mass tolerance was set to 5 ppm and MS/MS tolerance to 0.05 Da. The “match between runs” option was enabled in MaxQuant to transfer identifications made between runs on the basis of matching precursors with high mass accuracy. PSM and protein identifications were filtered using a target-decoy approach at a false discovery rate (FDR) of 1%.

### 2.6. RNA Extraction and RNA-Seq

RNA was extracted from lymphoblast cell lines using the Monarch Total RNA Miniprep Kit (NEB, Ipswich, MA, USA) and the manufacturer’s instructions for mammalian cells. Approximately 3 × 10^6^ lymphoblasts were harvested by centrifugation at 500× *g* for 5 min and resuspended in 300 µL of RNA lysis buffer. The sample was transferred to a gDNA removal column fitted to a microcentrifuge tube and spun at maximum speed for 30 s to collect any contaminating genomic DNA. The RNA in the flow-through was precipitated by the addition of 300 µL ethanol (Chem Supply, Port Adelaide, SA, Australia) and mixed by repeated pipetting. The suspension was transferred to an RNA purification column fitted to a collection tube and again spun at max speed for 30 s and the flow-through discarded. The RNA was washed by the addition of 500 µL RNA wash buffer and centrifugation, as described previously, and the flow-through was discarded. The sample was DNase-treated by the addition of 5 µL DNase I and 75 µL of DNase I reaction buffer to the column matrix and incubated at RT for 15 min. A 500 µL volume of RNA priming buffer was added to the column, centrifuged for 30 s at max speed, and the flow-through was discarded. The RNA in the column was washed twice with 500 µL of RNA wash buffer, followed by centrifugation as described previously. RNA was eluted by the addition of 50 µL nuclease-free water and centrifugation at max speed for 30 s. RNA was then stored at −80 °C before being sent to Novogene, Singapore on dry ice for mRNA sequencing and quantification using the Illumina NovaSeq 6000 platform and paired-end 150 bp reads.

### 2.7. Software Programs and Statistical Analysis

Proteomics data were initially analysed using the software Scaffold (Proteome Software, Portland, OR, USA). This validated the mass spectrometry data and produced a list of detected proteins. The mean fold-change differential expression of the proteins in the PD cohort compared to the healthy control cohort was calculated and statistical significance determined via *t*-tests. The data was then exported into Excel and the Benjamini–Hochberg false discovery correction was used to calculate modified *p* values (*q* values), using a threshold of 0.05 to detect significant differentially expressed proteins. Proteins not detected in both the HC and PD cohorts were excluded from further analysis.

Transcriptomic data was initially analysed by Novogene (Singapore), the company that performed the RNA-Seq experiments, and the fragments per kilobase of transcript sequence per million base pairs (FKPN) for each gene was calculated based on the length of the gene and reads-counts mapped to each gene. The read-counts were adjusted by the edgeR program package within Bioconductor in R (https://www.r-project.org/, accessed on 19 May 2022) through one scaling normalized factor [18,19,20]. The *p* values were corrected for false discovery using the Benjamini–Hochberg method, and the threshold for significantly differential expression was set at the adjusted *p*-value threshold of 0.05 and an absolute fold-change of 2. Transcripts not detected in both the HC and PD cohorts were excluded from further analysis.

For pathway analysis, the PANTHER over-representation tool (http://www.pantherdb.org, accessed on 19 May 2022) was used [21,22,23,24]. The proteins and transcripts were separated into lists containing significantly up- or down-regulated genes. Each list was entered into the PANTHER over-representation tool; binomial tests were selected as the statistical test and, if required, *p* values were corrected for false discovery using the Benjamini–Hochberg method. The list of all the transcripts and proteins detected in the entire experiment was uploaded as the reference list used by PANTHER to determine the number of “expected” pathway hits for a given number of transcripts or proteins.

To calculate the significance of the proportion of genes or transcripts which were enriched in either the upregulated or downregulated group, the proportion test in R was employed. To determine if the magnitude of the change of mitochondrial proteins was significant, a single sample *t*-test was employed in R.

## 3. Results

### 3.1. Global Changes in Parkinson Disease Proteomes and Transcriptomes

**Figure 1:**
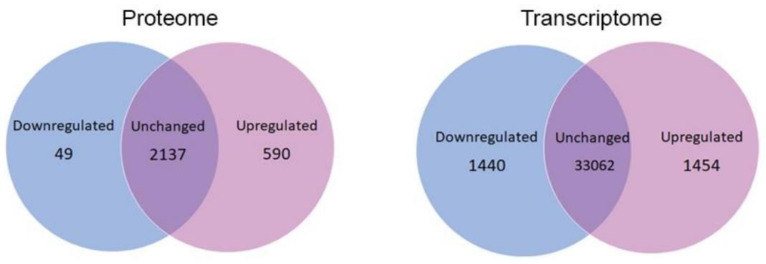
Global changes in protein and RNA expression.

Total protein and RNA were separately isolated from LCLs from idiopathic PD (iPD) and healthy control participants and sent for MS or RNA-Seq analysis. Mass Spectrometry identified a total of 2776 proteins, of which 49 were significantly downregulated and 590 were significantly upregulated after Benjamini–Hochberg correction for multiple comparisons (Figure 1). The transcriptomic analysis identified a total of 35,956 transcripts, of which 1440 were significantly downregulated and 1454 were significantly upregulated after Benjamini–Hochberg correction (Figure 1). Thus, there was almost no difference in the number of up- or downregulated transcripts, whereas a significantly higher number of proteins were upregulated compared to the number that were down-regulated. In fact, 21% of detected proteins were upregulated, whereas only 2% were downregulated (*p* < 2.2 × 10^−16^). By contrast, ca. 4% of transcripts were upregulated, and a similar percentage were downregulated. These differences show that the dysregulation of expression in iPD lymphoblasts occurs not only at the transcriptional level, but also at the translational level, and this is most pronounced in relation to the dramatic translational upregulation of up to ca. 20% of the proteome.

Venn diagrams depicting the number of differentially expressed gene products in lymphoblast cell lines generated from Parkinson’s Disease patients compared to Healthy Control Samples in whole cell proteomes and whole cell transcriptomes. Significantly more proteins were upregulated than were downregulated in the proteome (*p* < 2.2 × 10^−16^), whereas the numbers of up- and downregulated transcripts in the transcriptome were very similar (*p* = 0.81). The *p* values were calculated using a proportion test in R.

### 3.2. Cellular Component

As an initial step in our analysis, and to determine if proteins or transcripts associated with particular cell components were enriched in either the up- or downregulated groups, we employed the PANTHER over-representation tool.

### 3.3. Dysregulated Proteome

**Figure 2:**
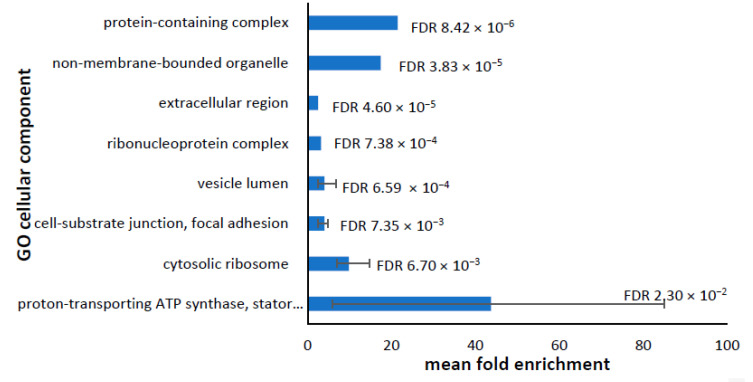
Most significantly enriched cellular components containing downregulated proteins. The Panther analysis of the annotated GO cellular components yielded a total of 30 cellular components that were significantly enriched in the downregulated proteins (Supplementary Information Table S1). Many components were represented by multiple GO terms—e.g., proton-transporting ATP synthase complex and mitochondrial proton-transporting ATP synthase complex—and were merged together. The merged GO cellular components were plotted against the mean fold enrichment, with lower and higher values represented by the error bars. The mean false discovery rate (FDR) for each merged GO cellular component is shown.

The cellular components with enriched representation amongst the downregulated proteins are shown in Figure 2. Several components were associated with the ribosome, suggesting a downregulation of protein synthesis. Other cellular components enriched in this group were the ATP synthase or Complex V of the oxidative phosphorylation (OXPHOS) pathway, which is surprising given the increase in ATP synthesis observed in functional studies with these cell lines [13]. There was also an enrichment of proteins associated with the cell surface and extracellular space, including focal adhesions, cell-substrate junctions, and extracellular vesicles.

**Figure 3:**
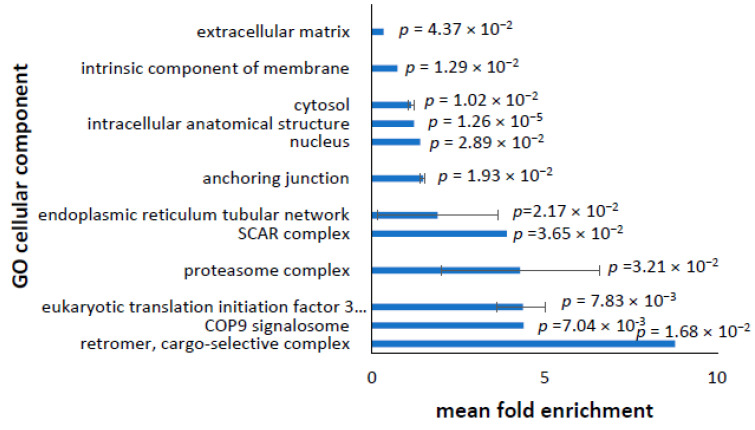
Most significantly enriched cellular components containing upregulated proteins. PanTable 26. cellular components that were significantly enriched in the upregulated proteins (Supplementary Information Table S2). Many components were represented by multiple GO terms and were merged together. The merged GO cellular components were plotted against the mean fold enrichment, with lower and higher values represented by the error bars. The mean *p* value for each merged cellular component, as calculated by t tests, are shown.

Amongst the upregulated proteins, the only cellular component that was significantly enriched was the cytosol, with a fold-change enrichment of 1.12 and an FDR *p*-value of 1.90 × 10^−^^2^. This suggests that the bulk of the upregulated proteins and processes were located within the cytosol rather than, for example, the nucleus or the mitochondria. To ensure that the FDR calculation was not too conservative, we repeated the analysis with no false discovery rate correction; this revealed 26 further cellular components that were enriched in upregulated proteins (Full list in Supplementary Information Table S2). Overlapping redundant terms were merged and terms which were not directly relevant to the cell type were removed—the remaining cell components are shown in Figure 3. The most significantly enriched upregulated cellular components, showing the highest fold-changes, play roles in translation initiation—which is interesting, as the most enriched downregulated proteins are associated with the ribosome, suggesting decreased protein production. This suggests that whilst the early stages of translation are upregulated, the latter stages are not. Translation initiation is generally considered to be the rate-limiting step in translation. It may also be that the activity or phosphorylation state of the initiation factors are altered so as to prevent rather than activate translation [25]. Proteins activated via stress to the cell, such as 4E-BP1 and S6K—both activated themselves by mTOR—are known to inactivate eukaryotic initiation factors, thereby reducing translation rates [26]. In support of this, increased phosphorylation of specific eukaryotic initiation factors has been observed in brain regions from PD patients [27], and impaired eIF signalling has been observed in PBMCs from sporadic and LRRK2 mutant patients [28]. There is an enrichment of the proteosome complex and components involved in protein secretion and recycling, such as the COP9 signalosome, ER network, retromer complex, and the SCAR complex, suggesting a greater turnover or salvaging of proteins and components. The proteosome complex may recycle the initiation factors that are inactivated and targeted for degradation.

### 3.4. Dysregulated Transcriptome

**Figure 4:**
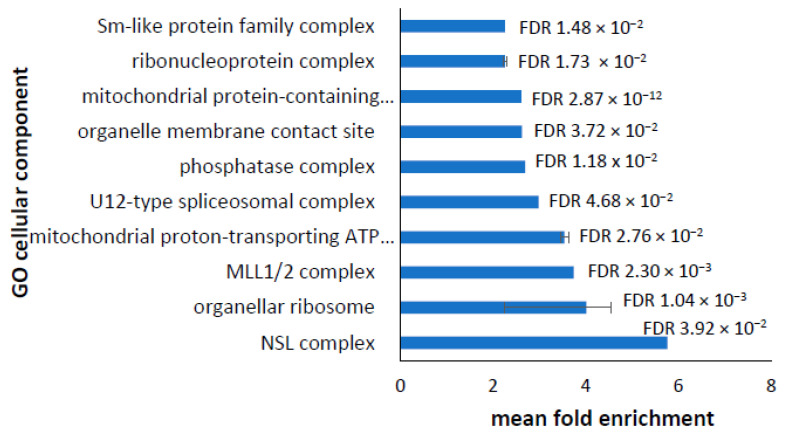
Most significantly enriched cellular components in downregulated transcripts. Panther analysis of annotated GO cellular components yielded a total of 101 cellular components that were significantly enriched in the downregulated transcripts (Supplementary Information Table S3). Many components were represented by multiple GO terms and were merged together. The merged GO cellular components were plotted against the mean fold enrichment, with lower and higher values represented by the error bars. The mean False Discovery Rates (FDR) are shown.

In the downregulated transcripts, the 10 most-enriched cellular components showing the greatest fold enrichment are as shown in Figure 4 and were largely representative of the full list (Supplementary Material Table S3). There is an enrichment of ribosomal subunits and the ATP synthase complex, both of which are in agreement with the proteomic data. There was also an enrichment of transcripts encoding proteins involved in RNA processing, histone methylation and acetylation, and the phosphatase complex. Additionally, the NonSpecific Lethal (NSL) complex, which is involved in upregulating gene expression, acetylation of histones, stabilizing microtubules, maintaining mitochondrial gene transcription, and facilitating the amino acid-dependent activation of TOR Complex I (TORC1) was downregulated [29]; this suggests the possibility that the reduced expression of NSL in the PD lymphoblasts may mediate the accompanying, general downregulation of genes involved in protein biosynthesis [29].

**Figure 5:**
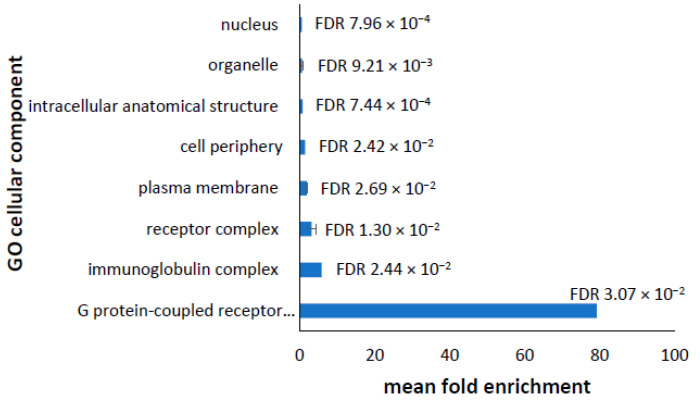
Most significantly enriched cellular components in upregulated transcripts. Panther analysis identified a total of 20 cellular components that were significantly enriched in the upregulated transcripts (Listed in Supplementary Information Table S4). GO cellular components represented by more than one GO term were merged together, reducing the list to eight. The mean fold enrichment of each cellular component is shown, with error bars representing higher and lower fold enrichments for the merged terms. False Discovery Rates (FDR) are shown for each cellular component.

In the upregulated transcripts, the most significantly enriched cellular components shown in Figure 5 are associated with the plasma membrane, receptor complexes, the immunoglobulin complex, and the nucleus. This shows some overlap with the enriched cellular components in the upregulated proteome and may also suggest an enrichment of receptor recycling and protein secretion.

### 3.5. Analysis of Altered GO Biological Processes in Differentially Expressed Transcripts and Proteins in PD Cell Lines

In order to gain an understanding of which biological processes were differentially regulated in the PD cell lines, the PANTHER over-representation tool was employed.

### 3.6. Dysregulated Proteome

**Figure 6:**
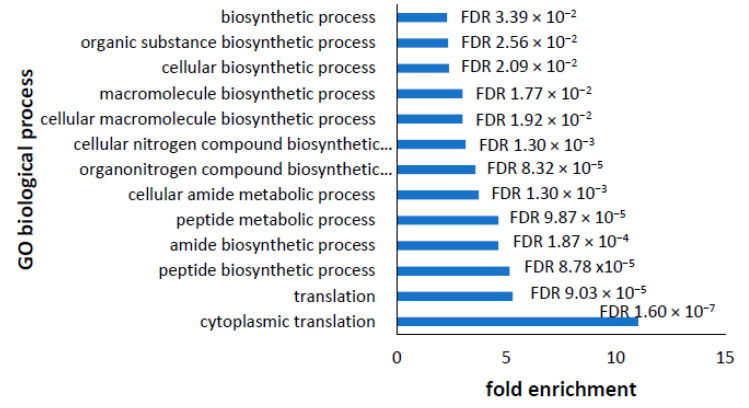
Enrichment of GO biological processes in significantly downregulated proteins. Panther pathway analysis of GO biological processes enriched in the downregulated proteins identified 13 processes all associated with protein biosynthesis. The GO biological process was plotted against the fold enrichment and the significance is shown for each as a False Discovery Rate (FDR) value.

The GO biological process annotations produced a list of GO biological processes which were over-represented in the up- or downregulated groups. In the downregulated proteins, a single major biological process was enriched—protein biosynthesis—and it was captured in a list of 13 significant, partly redundant GO terms Figure 6. This is in line with the cellular component and also with recent reports of a role for LRRK2 in directly phosphorylating components of the translation machinery and repressing global protein synthesis in both familial PD models and in fibroblasts from iPD patients [30].

**Figure 7:**
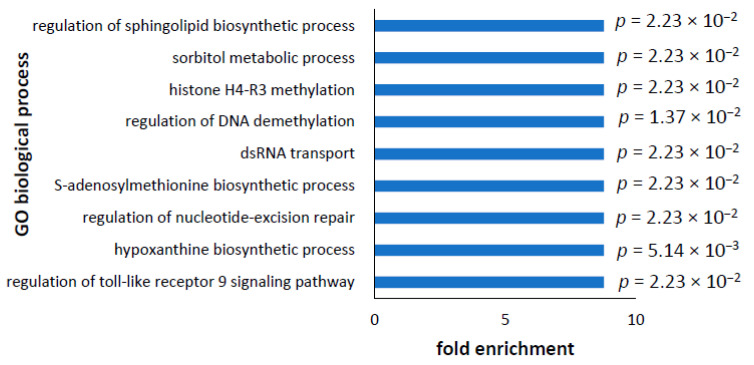
Most significantly enriched GO biological processes in upregulated proteins. The top 10 most enriched GO biological processes in the upregulated proteins were plotted against fold enrichment. The highest fold-change was 8.78, and this was the case for all of the top ten processes. In each case, all of the proteins identified for a biological process in the full reference list were also present in the upregulated proteins. The statistical significance (*p* value) as determined by *t*-tests varied depending on how many proteins were identified in the biological process. Redundant terms and processes not directly relevant to the cell type were removed from this list. The full list of biological processes can be found in Supplementary Material Table S5.

Amongst the many upregulated proteins, no biological processes were overrepresented, suggesting that the upregulation of such a large number of proteins was widespread across many biological processes identifiable by GO annotation. In view of this, we removed the false discovery rate correction, and this resulted in the identification of 440 processes—confirming that the upregulation of protein expression in PD lymphoblasts compared to controls was widespread across many biological processes and cellular functions. Some of these were not obviously relevant to the cell type we used, e.g., they had to do with axon or cardiac tissue development, and so were removed from the list below. In addition, many were repetitive and were merged together. The ten GO biological processes with the highest fold-changes that were directly relevant to the cell type are shown in Figure 7 and were associated with immune processes, DNA repair, biosynthetic pathways associated with nucleic acid and lipid synthesis, and post-translational modification. The full list of enriched, upregulated GO biological processes can be found in the Supplementary Material (Table S5) and were similar to the top ten—with the addition of signal transduction—including TOR signalling, cell death, cell cycle, ATP synthesis, and cell development. The enrichment of ATP synthesis in the upregulated proteins is in contrast to the enrichment of the ATP synthase complex in downregulated protein cellular components. How ATP synthesis could be enriched at the same time as proteins comprising the ATP synthase complex are downregulated was further investigated in the analysis of mitochondrial proteins in the section below.

### 3.7. Dysregulated Transcriptome

**Figure 8:**
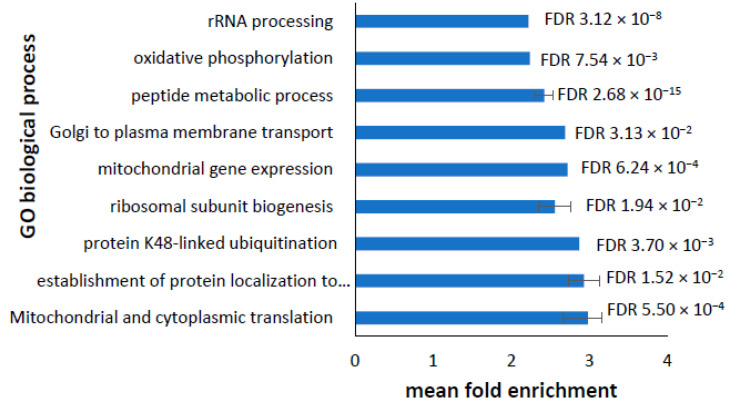
Most significantly enriched GO biological processes in downregulated transcripts. Panther pathway analysis was used to detect the enriched biological processes represented in the downregulated transcripts. Redundant overlapping terms were merged together and processes not directly relevant to the cell type were removed. The top 10 most enriched processes are depicted with their mean fold enrichment. Error bars represent the upper- and lower-fold enrichment values for the merged terms. Statistical significance is indicated by the false discovery rate (FDR)—for the merged terms, this is the mean FDR.

Amongst the downregulated transcripts, there were 184 GO biological processes identified as enriched (Supplementary Table S6). The large majority of these were to do with protein production, concordant with the observed enrichment of protein production pathways in the downregulated proteome. Reflective of this, the top ten downregulated processes represented in the transcriptome by the highest fold-change in expression were also associated with protein production (Figure 8). In the top ten, there was also an enrichment of oxidative phosphorylation and mitochondrial translation (Figure 8). This again highlights an anomaly in the representation of proteins and transcripts involved in oxidative phosphorylation and ATP synthesis, which will be discussed in detail in the next section.

**Figure 9:**
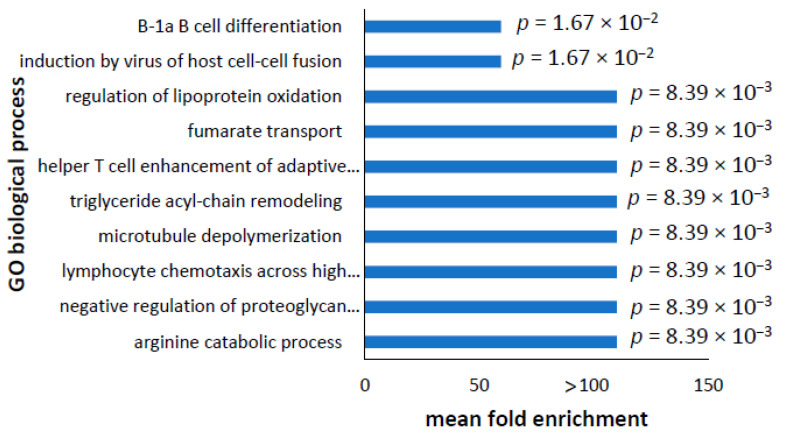
Most significantly enriched GO biological processes in upregulated transcripts. Using Panther pathway analysis in upregulated transcripts and with correction for false discovery rate, no GO biological processes were enriched. In case the FDR was too conservative and the biological processes were spread across many processes, the FDR correction was removed and the analysis repeated. This identified 606 GO biological processes—the full list is shown in Supplementary Material Table S7. Redundant terms were merged together and processes not directly relevant to the cell type were removed. The top 10 enriched biological processes are shown with their mean fold enrichment, and the significance determined by *t*-tests is indicated by the *p* value. Many enriched pathways had a fold-change of >100, and in these cases, had only one transcript identified as belonging to that pathway in both the full list of transcripts and the upregulated transcripts. Other enriched biological processes had a fold-change enrichment of 59.38 and had one of two identified transcripts in the full dataset that were present in the upregulated transcripts.

In the upregulated transcripts, the top 10 enriched processes with the highest fold-change were associated with amino acid and lipid catabolism, microtubule organisation, lymphocyte chemotaxis, fumarate transport, and immune processes (Figure 9). The full list of enriched GO biological processes in upregulated transcripts can be found in the Supplementary Material Table S7 and were largely the same as the top ten, with the addition of cell adhesion and migration processes and cell development and differentiation.

Combined, this data suggests that while some processes were concordant at the transcript and protein level (e.g., downregulation of protein biosynthetic machinery and upregulation of pathways for lipid and amino acid biosynthesis in both the transcriptome and the proteome), other processes were not. These discordant processes were particularly isolated to the upregulated proteins and transcripts, and indeed, this is where we found the greatest difference overall—more proteins were upregulated than downregulated in the proteome, whereas there were a similar number of mRNAs upregulated and downregulated in the transcriptome. An example of such differences is the enrichment of DNA repair processes and ATP synthesis in the upregulated proteome, but not the transcriptome.

In view of our previous functional analysis of these cell lines—showing an elevation of mitochondrial respiration in the PD cells in comparison to the healthy control cell lines [13]—and the surprising results above in regard to ATP synthesis production and ATP synthase components, we decided to investigate proteins and transcripts associated with the mitochondria in more detail.

### 3.8. Analysis of Mitochondrial Proteins

**Figure 10:**
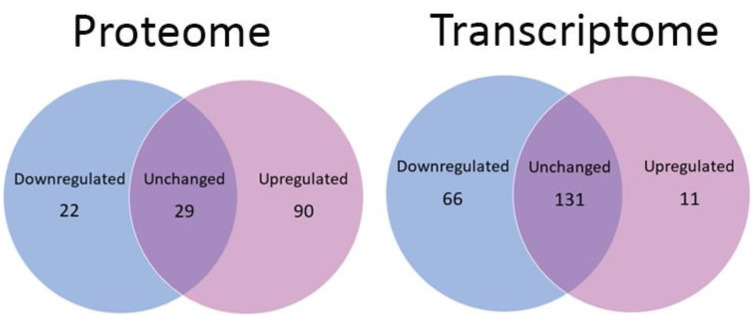
Changes in the abundance of mitochondrial transcripts and proteins. Venn diagrams depict the number of differentially expressed gene products associated with mitochondria in lymphoblast cell lines generated from Parkinson’s disease patients compared to healthy control samples in whole-cell proteomes and whole-cell transcriptomes. A significantly greater number of mitochondrial proteins were upregulated than downregulated in the proteome (2.2 × 10^−16^), whereas a significantly higher number of mitochondrial transcripts were downregulated in the transcriptome than were upregulated (*p* = 9.29 × 10^−12^). The *p* values were calculated using a proportion test in R.

To interrogate the mitochondrial pathways in more detail, the mitochondrial proteins and transcripts were identified from the entire list of proteins and transcripts. Proteins and transcripts had to be identified in at least five PD and five healthy control samples to be included. If the protein/transcript had a fold-change higher than 1.1—indicating a 10% increase in abundance—then they were classified as having a higher abundance, and conversely, if the protein/transcript had a fold-change lower than 0.9—indicating a 10% decrease in abundance—then they were classified as having a lower abundance. The abundances were averaged across samples from each group. The Venn diagrams in Figure 10 show that there were 141 mitochondrial proteins detected in at least five PD and control samples—22 of them were downregulated, whereas 90 were upregulated. In the whole-cell transcriptome, 208 mitochondrial transcripts were detected in at least five PD and control samples; of these, 66 were downregulated, while only 11 were upregulated. Thus, there was a significant global downregulation of mitochondrial transcripts, but a significant upregulation of mitochondrial proteins.

### 3.9. Dysregulated Mitochondrial Proteome

A full list of the mitochondrial proteins and mitochondrial transcripts and their abundances can be seen in Supplementary Figures S8 and S9, respectively. The proteins and transcripts were assigned to a functional group and the number of proteins and transcripts in each group was determined (Table 1 and Table 2, respectively). For the purposes of statistical analysis, any proteins/transcripts with a fold-change higher than 1 were classed as upregulated and, conversely, those with a fold-change lower than 1 were classified as downregulated. To assess the magnitude of the change, the fold-change was averaged for each protein/transcript in the up- or downregulated groups. To assess the significance of the overall fold-change, a single sample t-test was performed.

In view of the foregoing results revealing the downregulation of complex V, and previous reports that oxidative phosphorylation rates are elevated in many cellular PD models including lymphoblastoid cell lines [13,15,16,17], proteins involved in oxidative phosphorylation were investigated first. Based on the assumption that the individual proteins associated with particular respiratory complexes and their cognate transcripts should be coordinately regulated, we tested whether the expression of specific complexes were up- or downregulated. A higher proportion of proteins associated with complexes I–IV were upregulated than were downregulated, but this reached statistical significance only for Complex I—possibly due to the greater number of proteins detected in this complex (i.e., “sample size”). The extent of upregulation (assessed by fold-change) was greater than the reduction in the expression of downregulated proteins, and the magnitude of the overall increase in expression was significant for Complex I and IV, and approaching significance for Complex II. This was the opposite for Complex V, with a significant proportion of the individual proteins expressed at lower than control levels—but the overall magnitude of this change was not significant. In support of this finding of reduced Complex V expression and assembly in the PD cells, the protein OXA1L—which is involved in the assembly of complex V—was also reduced. By contrast, IF1—which, in its unphosphorylated state, inhibits ATP synthase—was significantly more highly expressed. The increased levels of complexes I, II, and IV in the iPD proteomes is consistent with our previously reported functional data showing an increase in mitochondrial respiration and no change in functionality of any of the complexes [13]. However, the reduced abundance of Complex V subunits in the proteome appears inconsistent with the elevated rates of OXPHOS and ATP steady state levels found previously. Furthermore, western blot analysis of a complex V subunit, ATP5A, previously showed an elevation in the abundance of this protein in PD lymphoblasts compared to a small control sample [13]—whereas in this study, its abundance was reduced by 15% (fold-change = 0.85) in the PD proteomes compared to those of a larger sample of healthy controls. We consider the proteomic result here to be more trustworthy because of the larger samples, the congruence of the result amongst multiple Complex V subunits, and the technically greater reliability of the proteomics approach compared to semiquantitative western blotting. That OXPHOS rates and ATP steady state levels are nonetheless elevated presumably results from the many factors controlling respiratory ATP synthesis in living cells, including not only the expression levels of the complexes, but also substrate supplies, cellular ATP demand, and regulatory signalling pathways. In support of increased mitochondrial respiration, there was an increase in the expression of TCA cycle proteins and mitochondrial import proteins (TIMM/TOMM complex)—both in the number of proteins upregulated and in the magnitude of the increase.

We also searched the datasets for proteins involved in or regulating mitochondrial protein expression and several key proteins were found to be increased in their abundance, including PRKAA1 (the catalytic subunit of AMPK) and LAMTOR—an activator of MAPK and mTORC1.

### 3.10. Dysregulated Transcriptome-Encoding Mitochondrial Proteins

In contrast to the upregulation of mitochondrial respiratory complexes in the proteome, transcripts for Complexes I–V were significantly downregulated, as were transcripts encoding mitochondrial transport (Table 2). Transcripts for enzymes in the TCA cycle, which provides electrons to the electron transport chain, were not significantly altered in abundance. These results suggest that mitochondrial electron transport proteins were downregulated at the transcript level but upregulated at the translational level. In contrast, Complex V was downregulated in both the transcriptome and the proteome, although the overall magnitude of this reduction was not significant in the proteome.

In addition to proteins directly involved in respiration (TCA cycle and OXPHOS complexes), we examined the presence in the dysregulated transcriptome of mitochondrial transport proteins in the SLC25 family (which transport ions and diverse metabolites across the mitochondrial inner membrane) and the TIMM/TOMM complexes, which import nuclear-encoded mitochondrial proteins. A significantly greater proportion of transcripts encoding these proteins were downregulated than upregulated, but the overall magnitude of this downregulation was significant only for the complexes involved in polypeptide import. In the case of the SLC25 transporters, this could suggest that their diverse transport activities are not and do not need to be coordinately regulated, so that some are upregulated and others are downregulated. Both of these groups were upregulated in the proteome, but in neither case did the overall change reach statistical significance (Table 1). This lack of statistical significance notwithstanding, these trends indicate that these additional groups of mitochondrial proteins may also be translationally upregulated in PD cells.

## 4. Discussion

Research is revealing that neurodegenerative diseases such as PD are complex, likely to be systemic, and involve disruption to key cellular processes such as mitochondrial function and proteasomal and lysosomal processes. They are affected by a multitude of factors—primarily age and a combination of genetics and environment. Improvements in omics technologies and their combined usage is useful for deciphering which pathways and processes are altered in PD and how their expression is regulated. Here, we used LCLs from PD patients and age- and sex-matched healthy controls and observed that a large number of transcripts and proteins were differentially regulated between the two cohorts. The detection and quantification of transcripts and proteins relies on different technologies, with RNA-Seq being more sensitive than MS, as evident by the detection of an order of magnitude more transcripts than proteins. Approximately 8% of the transcripts were differentially regulated, with an equal proportion of transcripts being up- and downregulated. This is in contrast to the proteins, with 23% being differentially regulated and the large majority of these upregulated. This suggests that in addition to the differences attributed to the sensitivity of the methods, there are likely to be differences due to post-transcriptional regulatory processes. A disconnect or decoupling between transcript and protein levels has been noted by others previously in relation to ageing and PD [31,32]. In some cases, the decoupling was reported to be specific to particular pathways, and in others, it was more global and occurred across the genome. Various models have been used including simple eukaryotic yeast models, post-mortem brain tissue, and cultured cell models, and this may account for the differences in identified pathways and proteins, as major differential expression patterns are expected to be cell type-specific as well as being potentially disease-specific.

In this study, we observed transcription–translation coupling of expression of genes involved in protein biosynthesis, as both transcripts and proteins belonging to this pathway were over-represented in the downregulated groups. Protein production has been reported by others to be downregulated in PD. Fibroblasts from iPD patients showed a 40% reduction in protein production [30] and post-mortem brain tissue display changes in the phosphorylation of eukaryotic translation initiation factors 2 and 2a (eEF2 and eIF2α), consistent with protein synthesis being repressed [33]. This global decrease in protein synthesis has recently been shown to be mediated by LRRK2 [30], as treatment of idiopathic PD and LRRK2 (G2019S) PD fibroblasts with LRRK2 inhibitors rescued the defect. This has also been shown in other PD models, including rats exposed to rotenone, where protein translation was decreased in the striatum and substantia nigra; this was reversed upon treatment with a LRRK2 inhibitor [30].

Protein translation is largely controlled by TORC1 [34] and in times of plentiful nutrients is activated to increase protein synthesis and allow the cell to increase its growth. We have assessed TORC1 activity in these LCL PD cell lines via a functional assay investigating the phosphorylation status of its downstream target 4E-BP1 (unpublished data) and found no significant difference in TORC1 activity between PD and HC cell lines. Regulation of TOR signalling was an enriched biological process in the upregulated proteins, and this was mainly due to the presence of two proteins: LAMTOR3 and PRKAA1. LAMTOR3 (Late endosomal/lysosomal adaptor and MAPK and mTOR activator 3) is a member of a scaffolding complex called Ragulator that anchors proteins to the lysosome, including TORC1 [35]. This brings TORC1 to the location where it can be activated by upstream proteins such as Rheb, suggesting that TORC1 may be more available for activation. The other subunits of this complex were not, however, significantly upregulated. The other protein in the TORC1 signalling pathway whose expression was upregulated was PRKAA1 (protein kinase AMP-activated catalytic subunit alpha 1), which encodes the catalytic subunit of a key protein involved in sensing and responding to energy stress AMP-activated protein kinase (AMPK). TORC1 is tightly regulated, and when growth conditions are not favourable, it is inactivated to reduce cell growth and increase autophagy. This inactivation can occur via AMPK [36,37]. In support of an upregulation of AMPK activity, our previous functional studies in these cell lines showed that AMPK is more highly activated in these PD cell lines [13]. This supports the functional data and suggests that AMPK is more active in these cell lines, inhibiting TORC1 and thereby repressing protein synthesis. In further support of downregulated TORC1 signalling, the components of the NSL (non-specific lethal) complex—which is involved in the activation of TORC1 signalling [29]—were enriched in the downregulated transcripts. Together, our data suggest that TORC1 signalling may be decreased in PD LCLs and that this results in global downregulation of protein synthesis.

Other biological processes that showed a transcription–translation coupling were immune processes, cell development, and biosynthetic pathways for lipids—particularly sphingolipids. These biological processes were enriched in both the upregulated proteins and transcripts.

An altered immune system has been implicated in both the susceptibility to PD and in the progression of the disease. Several studies have reported an increased susceptibility to PD in conjunction with autoimmune disorders such as thyroid diseases—Grave’s and Hashimoto’s [38]—and rheumatic diseases [39]. Autoimmune diseases share common altered pathways and proteins and GWAS studies have identified an overlap in regions linked to PD and to autoimmune disorders [40,41]. Neuroinflammation and an upregulated inflammatory response have been indicated in PD by the detection of more activated microglia [42]; increased levels of cytokines in the brain, CSF, and blood [43,44,45]; and increased levels of C-reactive protein (CRP) in serum and CSF, which is produced in response to proinflammatory cytokines and is expressed in the acute phase of inflammation [46]. The upregulation of immune processes has been noted in previous transcriptomic studies using blood and skin from iPD patients [8,47] and our results are in agreement with a heightened immune system and a systemic response.

Sphingolipids are the second most abundant group of lipids, second only to phospholipids [48], and play major roles within the cell. They are essential for maintaining cell structure, the binding of extracellular proteins, and microorganisms and numerous signal transduction pathways—including those regulating cell growth, endocytosis, differentiation, and responses to agonists [49]. Sphingolipids are regulated by metabolic requirements and their synthesis is inhibited by TORC1. They accumulate in the plasma membrane and in endosomes and are abundant in the CNS [50]. They have been implicated in several neurodegenerative diseases including PD. The first line of evidence which suggested the involvement of sphingolipids in PD came from yeast studies, which showed that the toxicity of α-synuclein was enhanced by the mutation of lipid elongase proteins involved in sphingolipid metabolism [51]. Since then, further support for a role of altered sphingolipid metabolism has emerged—for example, the most common risk factor for PD is mutations in glucocerebrosidase 1 (GBA1) [52], which is an enzyme involved in hydrolysing the sphingolipid glucocerebroside. Lipidomic studies from PD brains [53], PD plasma samples [54], and proteomic studies from PD plasma samples [54] have all shown dysregulated sphingolipid metabolism. Whilst the number and type of sphingolipids are diverse and complex, and we did not investigate this in further detail, it is clear that our data are in agreement with altered sphingolipid metabolism and altered TORC1 signalling. Lipidomic studies would be useful for characterising this further.

Unlike the downregulated proteome, which was dominated by pathways regulated at the transcriptional level, the upregulated proteome included major pathways not enriched in the upregulated transcriptome. This phenomenon of mRNA and protein decoupling has been noted in the aging brain, where a large number of proteins were differentially regulated, but the mRNA encoding these proteins was not [31]. The most significant difference was observed in mitochondrial oxidative phosphorylation and associated pathways, which largely showed enrichment in downregulated transcripts and upregulated proteins—with the exception of proteins encoding Complex V components. We have previously performed functional analyses with these cell lines and observed increased mitochondrial respiratory activity and an increase in ATP steady-state levels. This was not due to a functional difference in any of the complexes, but rather an upregulation of the system as a whole [13]. Whereas the levels of respiratory complexes I, III, and IV were higher in the PD lymphoblasts, as found previously using western blots of specific subunits [13], Complex V was significantly downregulated in both the transcriptome and the proteome.

A reduction in Complex V activity is supported by the decreased expression of OXA1L, which is required for the proper assembly of Complex V [55], and the increase in the protein ATPase Inhibitory Factor-1 (IF1). In its nonphosphorylated form, IF1 binds to and inhibits ATP synthase hydrolase and synthase activities. Its expression is tissue-specific and is regulated at the post-transcriptional level [56]. These two proteins affect the assembly of CV and its activity, respectively, but do not necessarily affect protein levels—which is what we measured here. Since our functional data revealed no functional impairment of Complex V, but elevated activity in PD LCLs [13] (and other cellular PD models [15,16,17]), the results here suggest that the elevated activity in intact cells was not a reflection of elevated expression, but might have been mediated by other factors.

It is known that Complex V activity is regulated by the cAMP–PKA pathway. PKA not only phosphorylates IF1, which would otherwise inhibit Complex V [57], but cAMP can also affect the organisation and activity of Complex V independently of IF1 [58]. De Rasmo et al. [58] showed in rat cells that a reduction in cAMP can lead to a reduction in the expression of ATP synthase subunits associated with the stator portion of the protein. This then leads to an instability and reduction of the oligomeric form of the complex, which affects the formation of cristae in the mitochondrial membrane and the efficiency of energy coupling, resulting in a membrane leak [58]. It is thus possible that the elevated activity of Complex V in functional studies in PD cells is a result of increased cAMP signalling.

Coupling of the proton gradient generated by Complexes I–IV to ATP synthesis by Complex V is important for the efficiency of ATP production. However, the proton is also utilised for other purposes aside from ATP production, including heat generation, fatty acid transport, mitochondrial protein import, ion transport across the mitochondrial membrane, and generation of reactive oxygen species (ROS) [59]. Although they were downregulated in the transcriptome, the proteins of the TIMM/TOMM complexes were upregulated in the proteomes of PD lymphoblasts. This is consistent with a general upregulation of mitochondrial activity and biogenesis in the PD cells.

The SLC25 transporters are a broad class of mitochondrial transporters that mediate the passage of diverse metabolites and ions across the mitochondrial inner membrane. They were downregulated in the transcriptome of PD cells, but in the proteome, similar proportions of SLC25 transporters were expressed at higher and lower levels than in healthy control cells. Thus, no global upregulation of mitochondrial SLC25 transporters was evident at the protein level. Amongst the upregulated proteins were carriers of dicarboxylate, citrate, aspartate/glutamate, and deoxynucleotide. These transporters are important for transporting intermediates for the TCA cycle and amino acid metabolism, transporting amino acids for mitochondrial protein synthesis, the export of citrate for the production of acetyl CoA, and can also transport adenine nucleotides for ATP synthesis [60]. The upregulation of these transporters therefore suggests an increased need for TCA cycle components and for the synthesis of lipids and amino acids. The downregulated mitochondrial transport proteins were mainly adenine nucleotide translocases—which are responsible for importing ADP into the mitochondria, where it is converted into ATP by Complex V [60]. The adenine nucleotides can also be used for the synthesis of mitochondrial RNA and DNA and other enzymatic reactions in the mitochondria. Furthermore, if adenine is lacking but fatty acids are present, then the transporters can act as uncoupling proteins [60].

Uncoupling proteins (UCP) uncouple the proton gradient generated by CI–CIV from ATP synthesis by Complex V. In addition to the adenine nucleotide translocases there are five UCPs within humans, and they are expressed differentially in tissues; in immune cells—including lymphoid cells—UCP2 is abundant, whereas other UCPs are not [61]. UCP2, in addition to its uncoupling role, is known to regulate lipid metabolism, generation of ROS, and inflammation. It plays important roles in the brain, regulating energy balance, inflammation, membrane potential, and cell death, and its dysregulation in PD results in increased neuronal cell death [62]. Some studies have shown that UCP2 is elevated in PD brains [63]. We searched for UCP2 in our data sets and identified it in the transcriptome, but not in the proteome, and its mRNA levels were unchanged in PD patients. UCP2, like other UCPs, has an unusually short half-life [64]—which may impede its detection in proteomic preparations. It remains possible that UCP2 may play a role in PD LCLs, which could be examined in future experiments.

ATP synthase activity can also be regulated post-translationally. This can occur by phosphorylation of the Complex V subunits, and the level of phosphorylation has been correlated with ATP synthase activity levels [65]. Other types of post-transcriptional regulation of Complex V have been reported in which the 3′UTR of a complex V subunit (subunit β) can be bound by regulatory proteins, resulting in reduced translation [66]. In addition, the ATP synthase Inhibitory Factor 1 (IF1) in its unphosphorylated form binds to and inhibits complex V—while when phosphorylated by cAMP, it dissociates from Complex V, releasing this inhibition [56,57].

It is not clear how ATP synthase is differentially regulated at the translation level in these PD LCLs, how it is regulated independently of the other OXPHOS complex subunits, or how—despite a reduced amount of protein—an increase in activity is evident. What is clear though, is that the regulation of ATP synthesis is complex, that it is finely controlled at multiple levels, and that its dysregulation is evident in PD. Further research into these mechanisms will be beneficial to our understanding of mitochondrial function in neurodegenerative diseases such as PD.

## 5. Conclusions

Our results using lymphoblastoid cell lines from Parkinson’s disease patients and healthy controls clearly show that a large number of genes are dysregulated. Many were differentially regulated at the transcriptional level, but there were in addition many that were upregulated post-transcriptionally. Our results demonstrate a global reduction in protein synthesis in PD lymphoblasts, an elevation of immune pathways, and an increase in sphingolipid metabolism. These data are in agreement with other PD studies using different PD tissue or cells and support the notion that PD is a systemic disease rather than being isolated to the brain. We also showed that—at least in LCLs from PD patients—the regulation of mitochondrial respiration is complex and involves translation regulation, and also likely post-translational regulation, which should be investigated in further studies. Our results suggest that LCLs, which are made from the most accessible tissue in the human body and are increasingly available in biobanks around the world, are useful for detecting disease-specific changes in protein expression. This approach has great potential for detecting differences in PD cohorts and in the early stages of disease progression.

## Figures and Tables

**Table 1:** Upregulation of Complexes I–IV and other mitochondrial respiration processes and downregulation of Complex V proteins.

Complex	Proteins Upregulated (Average Fold-Change)	Proteins Downregulated (Average Fold-Change)	Proportion Test (Significance *p*-Value)	*t*-Test (Significance *p*-Value)
Complex V	2 (1.65)	10 (0.85)	0.04	0.45
Complex IV	7 (1.70)	3 (0.86)	0.18	0.021
Complex III	6 (2.24)	3 (0.78)	0.35	0.11
Complex II	2 (1.26)	0	0.32	0.054
Complex I	19 (2.63)	7 (0.79)	0.0023	7.86 × 10^−4^
TCA	17 (1.8)	7 (0.92)	0.0094	0.0168
Mitochondrial transport (TIMM/TOMM complex)	7 (1.99)	2 (0.54)	0.059	0.0509
SLC25 transporters	6 (5.02)	5 (0.91)	1	0.103
Others	LAMTOR (1.88), PRKAA1 (2.4), OXA1L (0.69) (Assembly of CV & CIV), IF1 which inhibits ATP activity (4.0)			

**Table 2:** Downregulation of transcripts associated with mitochondrial respiration.

	Transcripts Upregulated (Average Fold-Change)	Transcripts Downregulated (Average Fold-Change)	Proportion Test (Significance *p*-Value)	*t*-Test (Significance *p*-Value)
Complex I	10 (1.04)	34 (0.91)	9.41 × 10^−7^	3.94 × 10^−6^
Complex II	2 (1.02)	6 (0.90)	0.13	9.01 × 10^−3^
Complex III	1 (1.10)	10 (0.89)	6.47 × 10^−4^	3.96 ×10^−3^
Complex IV	10 (1.07)	25 (0.90)	8.18 × 10^−4^	5.41 × 10^−3^
Complex V	3 (1.08)	16 (0.90)	9.89 × 10^−5^	8.41 × 10^−4^
TCA	7 (1.08)	11 (0.94)	0.32	0.40
SLC25 transporters	18 (1.34)	34 (0.91)	0.0033	0.83
TIMM/TOMM	4 (1.05)	19 (0.91)	7.11 × 10^−5^	1.50 × 10^−3^

## Data Availability

The raw proteomic and transcriptomic data has not been deposited in any publicly archived datasets, but all pathway analysis data and individual mitochondrial gene transcripts and proteins are provided in Supplementary Materials.

## Supplementary Materials

The following supporting information can be downloaded at: https://www.mdpi.com/article/10.3390/proteomes10020020/s1, Table S1: Enriched cellular components in downregulated proteins; Table S2: Enriched cellular components in upregulated proteins; Table S3: Enriched cellular components in downregulated transcripts; Table S4: Enriched cellular components in upregulated transcripts; Table S5: Enriched GO biological processes in upregulated proteins; Table S6: Enriched GO biological processes in downregulated transcripts; Table S7: Enriched GO biological processes in upregulated transcripts; Table S8: Mitochondrial proteins identified in the proteome; Table S9: Mitochondrial transcripts identified in the transcriptome.
