# Supplementary material for: Dysregulated Gene Expression in Lymphoblasts from Parkinson’s Disease
Source: Proteomes. 2022 Jun 1;10(2):20. doi: 10.3390/proteomes10020020 (PMC9230639; doi:10.3390/proteomes10020020)
Supplement: Supplementary file 1 [file proteomes-10-00020-s001.zip › proteomes-1695644-supplementary.pdf]

Table S1: Enriched cellular components in downregulated proteins

GO cellular component complete	Fold Enrichment	raw p-value	FDR
mitochondrial proton-transporting ATP synthase, stator stalk (GO:0000274)	85.02	2.67E-04	1.91E-02
proton-transporting ATP synthase, stator stalk (GO:0045265)	85.02	2.67E-04	1.82E-02
proton-transporting ATP synthase complex (GO:0045259)	21.26	3.97E-04	2.59E-02
mitochondrial proton-transporting ATP synthase complex (GO:0005753)	21.26	3.97E-04	2.48E-02
cytosolic large ribosomal subunit (GO:0022625)	14.79	6.33E-08	9.51E-06
cytosolic ribosome (GO:0022626)	13.16	1.04E-11	1.56E-08
cytosolic small ribosomal subunit (GO:0022627)	11.19	8.48E-05	6.37E-03
large ribosomal subunit (GO:0015934)	7.91	6.68E-06	7.17E-04
ribosomal subunit (GO:0044391)	7.57	8.40E-09	1.80E-06
ribosome (GO:0005840)	6.92	6.11E-09	1.53E-06
small ribosomal subunit (GO:0015935)	6.86	7.96E-04	3.99E-02
inner mitochondrial membrane protein complex (GO:0098800)	6	4.66E-04	2.80E-02
focal adhesion (GO:0005925)	4.83	1.50E-06	2.04E-04
cell-substrate junction (GO:0030055)	4.78	1.65E-06	2.06E-04
anchoring junction (GO:0070161)	3.84	7.78E-06	7.79E-04
secretory granule lumen (GO:0034774)	3.66	6.37E-04	3.54E-02
cytoplasmic vesicle lumen (GO:0060205)	3.64	6.59E-04	3.54E-02
vesicle lumen (GO:0031983)	3.63	6.82E-04	3.53E-02
ribonucleoprotein complex (GO:1990904)	3.17	7.86E-06	7.38E-04
extracellular exosome (GO:0070062)	2.68	1.55E-09	1.17E-06
extracellular vesicle (GO:1903561)	2.68	1.64E-09	8.20E-07
extracellular organelle (GO:0043230)	2.67	1.68E-09	6.32E-07
extracellular membrane-bounded organelle (GO:0065010)	2.67	1.68E-09	5.05E-07
extracellular space (GO:0005615)	2.4	2.90E-08	5.44E-06
cell junction (GO:0030054)	2.39	4.88E-04	2.82E-02
extracellular region (GO:0005576)	2.26	5.46E-08	9.12E-06
vesicle (GO:0031982)	1.94	2.63E-06	3.04E-04
intracellular non-membrane-bounded organelle (GO:0043232)	1.78	3.83E-05	3.20E-03
non-membrane-bounded organelle (GO:0043228)	1.78	3.83E-05	3.03E-03
protein-containing complex (GO:0032991)	1.68	8.42E-06	7.44E-04

Table S2: Enriched cellular components in upregulated proteins

GO cellular component complete	Fold Enrichment	raw p-value
retromer, cargo-selective complex (GO:0030906)	8.78	1.68E-02
cytosolic proteasome complex (GO:0031597)	6.58	2.72E-02
eukaryotic translation initiation factor 3 complex, eIF3m (GO:0071541)	5.01	1.99E-02
eukaryotic translation initiation factor 3 complex (GO:0005852)	5.01	9.85E-04
COP9 signalosome (GO:0008180)	4.39	7.04E-03
stereocilium bundle (GO:0032421)	4.39	2.74E-02

stereocilium (GO:0032420)	4.39	2.74E-02
eukaryotic 48S preinitiation complex (GO:0033290)	4.39	3.63E-03
SCAR complex (GO:0031209)	3.9	3.65E-02
eukaryotic 43S preinitiation complex (GO:0016282)	3.84	6.39E-03
endoplasmic reticulum tubular network (GO:0071782)	3.66	2.40E-02
translation preinitiation complex (GO:0070993)	3.61	8.24E-03
motile cilium (GO:0031514)	2.51	2.60E-02
proteasome complex (GO:0000502)	1.99	3.70E-02
focal adhesion (GO:0005925)	1.53	1.24E-02
cell-substrate junction (GO:0030055)	1.52	1.66E-02
anchoring junction (GO:0070161)	1.39	2.89E-02
cytosol (GO:0005829)	1.21	1.26E-05
nucleus (GO:0005634)	1.1	3.77E-02
cytoplasm (GO:0005737)	1.05	2.04E-02
intracellular anatomical structure (GO:0005622)	1.04	4.69E-04
intrinsic component of membrane (GO:0031224)	0.73	1.34E-02
integral component of membrane (GO:0016021)	0.73	1.24E-02
extracellular matrix (GO:0031012)	0.33	4.34E-02
external encapsulating structure (GO:0030312)	0.32	4.40E-02
blood microparticle (GO:0072562)	0.15	2.73E-02
endoplasmic reticulum protein-containing complex (GO:0140534)	0.14	1.93E-02

Table S3:Enriched cellular components in downregulated transcripts

GO cellular component complete	Fold Enrichment	raw p-value	FDR
NSL complex (GO:0044545)	5.76	2.01E-03	3.92E-02
organellar large ribosomal subunit (GO:0000315)	4.55	4.44E-10	2.19E-08
mitochondrial large ribosomal subunit (GO:0005762)	4.55	4.44E-10	2.13E-08
organellar ribosome (GO:0000313)	4.41	1.46E-14	1.31E-12
mitochondrial ribosome (GO:0005761)	4.41	1.46E-14	1.25E-12
organellar small ribosomal subunit (GO:0000314)	4.36	1.47E-05	5.56E-04
mitochondrial small ribosomal subunit (GO:0005763)	4.36	1.47E-05	5.45E-04
preribosome, large subunit precursor (GO:0030687)	4.2	4.40E-05	1.58E-03
cytosolic small ribosomal subunit (GO:0022627)	4.13	1.02E-07	4.27E-06
small ribosomal subunit (GO:0015935)	4.09	1.76E-11	9.37E-10
ribosomal subunit (GO:0044391)	4.03	1.74E-25	3.42E-23
large ribosomal subunit (GO:0015934)	3.99	9.13E-16	8.99E-14
MLL1/2 complex (GO:0044665)	3.74	6.94E-05	2.32E-03
MLL1 complex (GO:0071339)	3.74	6.94E-05	2.28E-03
cytosolic ribosome (GO:0022626)	3.66	8.40E-13	5.52E-11
mitochondrial proton-transporting ATP synthase complex (GO:0005753)	3.63	1.08E-03	2.38E-02
ribosome (GO:0005840)	3.61	1.35E-25	2.96E-23
cytosolic large ribosomal subunit (GO:0022625)	3.58	1.70E-07	6.96E-06
proton-transporting ATP synthase complex (GO:0045259)	3.45	1.50E-03	3.14E-02
U12-type spliceosomal complex (GO:0005689)	2.98	2.42E-03	4.68E-02
protein serine/threonine phosphatase complex (GO:0008287)	2.69	4.61E-04	1.18E-02

phosphatase complex (GO:1903293)	2.69	4.61E-04	1.17E-02
organelle membrane contact site (GO:0044232)	2.62	1.87E-03	3.72E-02
mitochondrial protein-containing complex (GO:0098798)	2.61	3.49E-14	2.87E-12
ribonucleoprotein complex (GO:1990904)	2.3	1.11E-24	1.83E-22
Sm-like protein family complex (GO:0120114)	2.26	6.48E-04	1.48E-02
preribosome (GO:0030684)	2.24	5.25E-04	1.29E-02
small nuclear ribonucleoprotein complex (GO:0030532)	2.23	1.70E-03	3.46E-02
protein acetyltransferase complex (GO:0031248)	2.22	6.17E-04	1.48E-02
acetyltransferase complex (GO:1902493)	2.22	6.17E-04	1.47E-02
methyltransferase complex (GO:0034708)	2.2	4.04E-04	1.08E-02
histone acetyltransferase complex (GO:0000123)	2.19	1.61E-03	3.34E-02
mitochondrial matrix (GO:0005759)	2.07	3.57E-13	2.70E-11
U2-type spliceosomal complex (GO:0005684)	2.06	1.23E-03	2.65E-02
mitochondrial inner membrane (GO:0005743)	2.01	3.17E-12	1.83E-10
inner mitochondrial membrane protein complex (GO:0098800)	1.96	2.52E-04	7.40E-03
organelle inner membrane (GO:0019866)	1.91	1.64E-11	8.98E-10
cullin-RING ubiquitin ligase complex (GO:0031461)	1.86	2.54E-04	7.37E-03
mitochondrial envelope (GO:0005740)	1.83	7.08E-14	5.58E-12
mitochondrial membrane (GO:0031966)	1.82	9.29E-13	5.91E-11
trans-Golgi network (GO:0005802)	1.71	1.51E-04	4.71E-03
intracellular protein-containing complex (GO:0140535)	1.7	4.95E-10	2.32E-08
transferase complex (GO:1990234)	1.68	1.00E-09	4.60E-08
envelope (GO:0031975)	1.62	4.14E-13	2.92E-11
organelle envelope (GO:0031967)	1.62	4.14E-13	2.81E-11
ubiquitin ligase complex (GO:0000151)	1.62	3.29E-04	9.26E-03
ribonucleoprotein granule (GO:0035770)	1.57	1.93E-03	3.81E-02
mitochondrion (GO:0005739)	1.57	5.05E-15	4.74E-13
catalytic complex (GO:1902494)	1.57	1.91E-12	1.14E-10
Golgi apparatus subcompartment (GO:0098791)	1.54	3.31E-04	9.19E-03
intrinsic component of organelle membrane (GO:0031300)	1.52	3.41E-04	9.33E-03
nuclear protein-containing complex (GO:0140513)	1.47	6.62E-08	2.84E-06
integral component of organelle membrane (GO:0031301)	1.46	1.67E-03	3.42E-02
nucleoplasm (GO:0005654)	1.41	6.95E-23	9.78E-21
nuclear lumen (GO:0031981)	1.39	1.70E-23	2.57E-21
centrosome (GO:0005813)	1.38	7.84E-04	1.78E-02
organelle lumen (GO:0043233)	1.38	6.79E-30	2.67E-27
intracellular organelle lumen (GO:0070013)	1.38	6.79E-30	2.23E-27
membrane-enclosed lumen (GO:0031974)	1.38	6.79E-30	1.91E-27
organelle subcompartment (GO:0031984)	1.34	6.37E-06	2.51E-04
nucleolus (GO:0005730)	1.32	3.66E-04	9.89E-03
protein-containing complex (GO:0032991)	1.32	3.00E-20	3.69E-18
microtubule organizing center (GO:0005815)	1.31	1.24E-03	2.64E-02
organelle membrane (GO:0031090)	1.3	1.36E-11	7.63E-10
endoplasmic reticulum membrane (GO:0005789)	1.29	4.36E-04	1.15E-02
nuclear outer membrane-endoplasmic reticulum membrane network (GO:0042175)	1.29	4.46E-04	1.16E-02
endoplasmic reticulum subcompartment (GO:0098827)	1.29	5.36E-04	1.30E-02

cytosol (GO:0005829)	1.29	4.61E-17	5.34E-15
nucleus (GO:0005634)	1.27	4.47E-25	8.01E-23
intracellular membrane-bounded organelle (GO:0043231)	1.21	1.04E-43	2.06E-40
bounding membrane of organelle (GO:0098588)	1.2	6.44E-04	1.49E-02
intracellular organelle (GO:0043229)	1.17	2.20E-35	2.17E-32
membrane-bounded organelle (GO:0043227)	1.16	3.05E-33	2.00E-30
organelle (GO:0043226)	1.13	6.04E-28	1.49E-25
cytoplasm (GO:0005737)	1.13	5.35E-17	5.86E-15
intracellular non-membrane-bounded organelle (GO:0043232)	1.13	1.35E-04	4.35E-03
non-membrane-bounded organelle (GO:0043228)	1.13	1.35E-04	4.28E-03
intracellular anatomical structure (GO:0005622)	1.13	7.57E-33	3.73E-30
extracellular space (GO:0005615)	0.86	1.05E-03	2.35E-02
extracellular region (GO:0005576)	0.83	2.09E-05	7.63E-04
integral component of membrane (GO:0016021)	0.79	2.06E-10	1.04E-08
intrinsic component of membrane (GO:0031224)	0.79	8.02E-11	4.16E-09
plasma membrane bounded cell projection (GO:0120025)	0.78	5.81E-05	2.01E-03
cell projection (GO:0042995)	0.77	1.17E-05	4.50E-04
neuron projection (GO:0043005)	0.74	1.64E-04	5.04E-03
plasma membrane (GO:0005886)	0.73	2.82E-16	2.92E-14
cell periphery (GO:0071944)	0.71	6.59E-21	8.66E-19
cell surface (GO:0009986)	0.65	6.56E-05	2.23E-03
plasma membrane protein complex (GO:0098797)	0.6	2.05E-04	6.11E-03
external side of plasma membrane (GO:0009897)	0.59	1.41E-03	2.98E-02
intrinsic component of plasma membrane (GO:0031226)	0.56	1.36E-12	8.35E-11
integral component of plasma membrane (GO:0005887)	0.54	4.11E-13	3.00E-11
receptor complex (GO:0043235)	0.5	5.72E-05	2.01E-03
ion channel complex (GO:0034702)	0.45	3.00E-04	8.57E-03
cation channel complex (GO:0034703)	0.4	6.22E-04	1.46E-02
motile cilium (GO:0031514)	0.4	4.92E-04	1.23E-02
extracellular matrix (GO:0031012)	0.38	9.87E-09	4.32E-07
external encapsulating structure (GO:0030312)	0.38	9.11E-09	4.08E-07
collagen-containing extracellular matrix (GO:0062023)	0.38	5.51E-07	2.22E-05
9+2 motile cilium (GO:0097729)	0.32	1.74E-03	3.50E-02
9+0 non-motile cilium (GO:0097731)	0.27	1.09E-03	2.39E-02
photoreceptor cell cilium (GO:0097733)	0.15	1.70E-04	5.16E-03

Table S4:Enriched cellular components in upregulated transcripts

**GO cellular component complete**	**Fold Enrichment**	**FDR**
G protein-coupled receptor heterodimeric complex (GO:0038039)	79.17	3.07E-02
immunoglobulin complex (GO:0019814)	5.85	2.44E-02
plasma membrane signaling receptor complex (GO:0098802)	5.32	1.29E-02
receptor complex (GO:0043235)	4.29	7.60E-04
integral component of plasma membrane (GO:0005887)	2.13	2.52E-02
intrinsic component of plasma membrane (GO:0031226)	2.03	3.19E-02
plasma membrane (GO:0005886)	1.49	2.35E-02
cell periphery (GO:0071944)	1.46	2.42E-02

intracellular anatomical structure (GO:0005622)	0.78	7.44E-04
organelle (GO:0043226)	0.74	2.04E-04
intracellular organelle (GO:0043229)	0.72	3.32E-04
membrane-bounded organelle (GO:0043227)	0.66	2.53E-06
intracellular membrane-bounded organelle (GO:0043231)	0.62	4.34E-06
organelle lumen (GO:0043233)	0.56	2.37E-02
intracellular organelle lumen (GO:0070013)	0.56	2.18E-02
membrane-enclosed lumen (GO:0031974)	0.56	2.03E-02
nucleus (GO:0005634)	0.55	7.96E-04
nuclear lumen (GO:0031981)	0.5	2.36E-02
nucleoplasm (GO:0005654)	0.45	1.54E-02
organelle membrane (GO:0031090)	0.38	7.35E-03

Table S5: Enriched GO biological processes in upregulated proteins

GO biological process complete	Fold Change	p value
axonal fasciculation (GO:0007413)	8.78	2.23E-02
positive regulation of toll-like receptor 9 signaling pathway (GO:0034165)	8.78	2.23E-02
regulation of toll-like receptor 9 signaling pathway (GO:0034163)	8.78	2.23E-02
hypoxanthine biosynthetic process (GO:0046101)	8.78	5.14E-03
hypoxanthine metabolic process (GO:0046100)	8.78	5.14E-03
regulation of nucleotide-excision repair (GO:2000819)	8.78	2.23E-02
cardiac epithelial to mesenchymal transition (GO:0060317)	8.78	2.23E-02
positive regulation of dopamine receptor signaling pathway (GO:0060161)	8.78	2.23E-02
regulation of dopamine receptor signaling pathway (GO:0060159)	8.78	2.23E-02
S-adenosylmethionine biosynthetic process (GO:0006556)	8.78	2.23E-02
dsRNA transport (GO:0033227)	8.78	2.23E-02
regulation of ceramide biosynthetic process (GO:2000303)	8.78	2.23E-02
positive regulation of synaptic vesicle exocytosis (GO:2000302)	8.78	2.23E-02
positive regulation of DNA demethylation (GO:1901537)	8.78	2.23E-02
regulation of DNA demethylation (GO:1901535)	8.78	5.14E-03
histone H4-R3 methylation (GO:0043985)	8.78	2.23E-02
sorbitol metabolic process (GO:0006060)	8.78	2.23E-02
regulation of sphingolipid biosynthetic process (GO:0090153)	8.78	2.23E-02
neuron projection fasciculation (GO:0106030)	8.78	2.23E-02
positive regulation of neurotrophin TRK receptor signaling pathway (GO:0051388)	8.78	2.23E-02
regulation of neurotrophin TRK receptor signaling pathway (GO:0051386)	8.78	2.23E-02
positive regulation of protein localization to endoplasmic reticulum (GO:1905552)	8.78	2.23E-02
regulation of protein localization to endoplasmic reticulum (GO:1905550)	8.78	2.23E-02
negative regulation of translation in response to stress (GO:0032055)	8.78	2.23E-02
positive regulation of mitochondrial ATP synthesis coupled electron transport (GO:1905448)	8.78	2.23E-02
protein localization to nuclear inner membrane (GO:0036228)	8.78	2.23E-02
negative regulation of granulocyte differentiation (GO:0030853)	8.78	2.23E-02
histone H2B conserved C-terminal lysine deubiquitination (GO:0035616)	8.78	2.23E-02
positive regulation of metallopeptidase activity (GO:1905050)	8.78	2.23E-02

regulation of membrane lipid metabolic process (GO:1905038)	8.78	2.23E-02
hypoxanthine salvage (GO:0043103)	8.78	2.23E-02
histone arginine methylation (GO:0034969)	8.78	5.14E-03
negative regulation of IRE1-mediated unfolded protein response (GO:1903895)	8.78	2.23E-02
peptidyl-arginine omega-N-methylation (GO:0035247)	8.78	5.14E-03
peptidyl-arginine methylation, to asymmetrical-dimethyl arginine (GO:0019919)	8.78	2.23E-02
regulation of membrane tubulation (GO:1903525)	8.78	2.23E-02
fructose biosynthetic process (GO:0046370)	8.78	2.23E-02
L-kynurenine catabolic process (GO:0097053)	8.78	2.23E-02
T-helper 17 cell lineage commitment (GO:0072540)	8.78	2.23E-02
T-helper 17 cell differentiation (GO:0072539)	8.78	2.23E-02
formaldehyde catabolic process (GO:0046294)	8.78	2.23E-02
formaldehyde metabolic process (GO:0046292)	8.78	2.23E-02
protein ufmylation (GO:0071569)	7.31	6.96E-04
protein K69-linked ufmylation (GO:1990592)	7.02	2.77E-03
protein polyufmylation (GO:1990564)	7.02	2.77E-03
positive regulation of gamma-delta T cell differentiation (GO:0045588)	6.58	1.12E-02
regulation of gamma-delta T cell differentiation (GO:0045586)	6.58	1.12E-02
T-helper cell lineage commitment (GO:0002295)	6.58	1.12E-02
peptidyl-arginine methylation (GO:0018216)	6.58	1.12E-02
CD4-positive, alpha-beta T cell lineage commitment (GO:0043373)	6.58	1.12E-02
CD4-positive or CD8-positive, alpha-beta T cell lineage commitment (GO:0043369)	6.58	1.12E-02
peptidyl-arginine N-methylation (GO:0035246)	6.58	1.12E-02
positive regulation of gamma-delta T cell activation (GO:0046645)	6.58	1.12E-02
regulation of gamma-delta T cell activation (GO:0046643)	6.58	1.12E-02
protein hexamerization (GO:0034214)	5.85	4.66E-02
adenine biosynthetic process (GO:0046084)	5.85	4.66E-02
adenine metabolic process (GO:0046083)	5.85	4.66E-02
negative regulation of T cell mediated cytotoxicity (GO:0001915)	5.85	4.66E-02
positive regulation of neutrophil activation (GO:1902565)	5.85	4.66E-02
definitive hemopoiesis (GO:0060216)	5.85	4.66E-02
common-partner SMAD protein phosphorylation (GO:0007182)	5.85	4.66E-02
regulation of glycogen catabolic process (GO:0005981)	5.85	4.66E-02
positive regulation of intracellular estrogen receptor signaling pathway (GO:0033148)	5.85	4.66E-02
7-methylguanosine mRNA capping (GO:0006370)	5.85	4.66E-02
peptidyl-glutamic acid modification (GO:0018200)	5.85	4.66E-02
negative regulation of dendrite development (GO:2000171)	5.85	4.66E-02
histone H3 acetylation (GO:0043966)	5.85	4.66E-02
proteasome regulatory particle assembly (GO:0070682)	5.85	4.66E-02
hexitol metabolic process (GO:0006059)	5.85	4.66E-02
negative regulation of membrane permeability (GO:1905709)	5.85	4.66E-02
AMP salvage (GO:0044209)	5.85	4.66E-02
cellular response to leucine starvation (GO:1990253)	5.85	4.66E-02
regulation of mitochondrial ATP synthesis coupled electron transport (GO:1905446)	5.85	4.66E-02

positive regulation of potassium ion transmembrane transporter activity (GO:1901018)	5.85	4.66E-02
positive regulation of isotype switching to IgG isotypes (GO:0048304)	5.85	4.66E-02
regulation of isotype switching to IgG isotypes (GO:0048302)	5.85	4.66E-02
kynurenine metabolic process (GO:0070189)	5.85	4.66E-02
RNA capping (GO:0036260)	5.85	4.66E-02
negative regulation of mitochondrial membrane permeability (GO:0035795)	5.85	4.66E-02
7-methylguanosine RNA capping (GO:0009452)	5.85	4.66E-02
regulation of myofibroblast differentiation (GO:1904760)	5.85	4.66E-02
monoubiquitinated protein deubiquitination (GO:0035520)	5.85	4.66E-02
fatty acid homeostasis (GO:0055089)	5.85	4.66E-02
motor behavior (GO:0061744)	5.85	4.66E-02
purine nucleobase salvage (GO:0043096)	5.85	4.66E-02
histone-serine phosphorylation (GO:0035404)	5.85	4.66E-02
purine nucleoside triphosphate catabolic process (GO:0009146)	5.85	4.66E-02
dendrite extension (GO:0097484)	5.85	4.66E-02
maintenance of protein location in mitochondrion (GO:0072656)	5.85	4.66E-02
regulation of glial cell apoptotic process (GO:0034350)	5.85	4.66E-02
glial cell apoptotic process (GO:0034349)	5.85	4.66E-02
L-kynurenine metabolic process (GO:0097052)	5.85	4.66E-02
cerebral cortex neuron differentiation (GO:0021895)	5.85	4.66E-02
regulation of vacuolar transport (GO:1903335)	5.85	4.66E-02
T-helper 17 type immune response (GO:0072538)	5.85	4.66E-02
regulation of late endosome to lysosome transport (GO:1902822)	5.85	4.66E-02
alpha-beta T cell lineage commitment (GO:0002363)	5.27	2.01E-02
glycogen catabolic process (GO:0005980)	5.27	2.01E-02
viral translational termination-reinitiation (GO:0075525)	5.27	2.01E-02
negative regulation of protein kinase activity by regulation of protein phosphorylation (GO:0044387)	5.27	2.01E-02
regulation of stem cell population maintenance (GO:2000036)	5.27	2.01E-02
Golgi disassembly (GO:0090166)	5.27	2.01E-02
cellular polysaccharide catabolic process (GO:0044247)	5.27	2.01E-02
regulation of endodeoxyribonuclease activity (GO:0032071)	5.27	2.01E-02
protein deneddylation (GO:0000338)	5.27	1.13E-03
nuclear membrane disassembly (GO:0051081)	5.27	2.01E-02
polysaccharide catabolic process (GO:0000272)	5.27	2.01E-02
SCF-dependent proteasomal ubiquitin-dependent protein catabolic process (GO:0031146)	5.27	2.01E-02
glucan catabolic process (GO:0009251)	5.27	2.01E-02
deoxyribonucleoside triphosphate catabolic process (GO:0009204)	5.27	2.01E-02
nucleoside triphosphate catabolic process (GO:0009143)	5.27	2.01E-02
membrane disassembly (GO:0030397)	5.27	2.01E-02
cerebral cortex radially oriented cell migration (GO:0021799)	5.01	8.92E-03
telencephalon glial cell migration (GO:0022030)	5.01	8.92E-03
CD4-positive, alpha-beta T cell differentiation involved in immune response (GO:0002294)	5.01	8.92E-03
alpha-beta T cell differentiation involved in immune response (GO:0002293)	5.01	8.92E-03
alpha-beta T cell activation involved in immune response (GO:0002287)	5.01	8.92E-03

CD4-positive, alpha-beta T cell differentiation (GO:0043367)	5.01	8.92E-03
reticulophagy (GO:0061709)	5.01	8.92E-03
T-helper cell differentiation (GO:0042093)	5.01	8.92E-03
cerebral cortex radial glia-guided migration (GO:0021801)	5.01	8.92E-03
formation of cytoplasmic translation initiation complex (GO:0001732)	4.68	3.86E-04
cerebral cortex cell migration (GO:0021795)	4.39	1.39E-02
positive regulation of neutrophil migration (GO:1902624)	4.39	1.39E-02
positive regulation of B cell differentiation (GO:0045579)	4.39	3.21E-02
T cell lineage commitment (GO:0002360)	4.39	3.21E-02
negative regulation of posttranscriptional gene silencing (GO:0060149)	4.39	3.21E-02
positive regulation of mRNA binding (GO:1902416)	4.39	3.21E-02
regulation of mRNA binding (GO:1902415)	4.39	1.39E-02
T cell differentiation involved in immune response (GO:0002292)	4.39	1.39E-02
receptor catabolic process (GO:0032801)	4.39	3.21E-02
peptidyl-arginine modification (GO:0018195)	4.39	3.21E-02
protein K48-linked deubiquitination (GO:0071108)	4.39	3.21E-02
purine nucleotide salvage (GO:0032261)	4.39	3.21E-02
photoperiodism (GO:0009648)	4.39	3.21E-02
entrainment of circadian clock by photoperiod (GO:0043153)	4.39	3.21E-02
regulation of IRE1-mediated unfolded protein response (GO:1903894)	4.39	3.21E-02
negative regulation of gene silencing by RNA (GO:0060967)	4.39	3.21E-02
determination of adult lifespan (GO:0008340)	4.39	3.21E-02
microtubule anchoring (GO:0034453)	4.39	3.21E-02
cellular carbohydrate catabolic process (GO:0044275)	4.05	4.12E-03
nuclear migration (GO:0007097)	3.99	9.11E-03
positive T cell selection (GO:0043368)	3.99	9.11E-03
multicellular organism aging (GO:0010259)	3.9	2.04E-02
positive regulation of cell cycle G2/M phase transition (GO:1902751)	3.9	2.04E-02
telencephalon cell migration (GO:0022029)	3.9	2.04E-02
B cell homeostasis (GO:0001782)	3.9	2.04E-02
positive regulation of organ growth (GO:0046622)	3.9	2.04E-02
forebrain cell migration (GO:0021885)	3.9	2.04E-02
positive regulation of heart growth (GO:0060421)	3.76	4.69E-02
cellular response to aldehyde (GO:0110096)	3.76	4.69E-02
regulation of neutrophil activation (GO:1902563)	3.76	4.69E-02
endoplasmic reticulum tubular network organization (GO:0071786)	3.76	4.69E-02
cellular response to BMP stimulus (GO:0071773)	3.76	4.69E-02
response to BMP (GO:0071772)	3.76	4.69E-02
positive regulation of granulocyte chemotaxis (GO:0071624)	3.76	4.69E-02
myeloid dendritic cell activation (GO:0001773)	3.76	4.69E-02
regulation of production of small RNA involved in gene silencing by RNA (GO:0070920)	3.76	4.69E-02
positive regulation of neutrophil chemotaxis (GO:0090023)	3.76	4.69E-02
mitochondrial fragmentation involved in apoptotic process (GO:0043653)	3.76	4.69E-02
regulation of deoxyribonuclease activity (GO:0032070)	3.76	4.69E-02
entrainment of circadian clock (GO:0009649)	3.76	4.69E-02
Golgi inheritance (GO:0048313)	3.76	4.69E-02
organelle inheritance (GO:0048308)	3.76	4.69E-02
negative regulation of plasma membrane bounded cell projection assembly (GO:0120033)	3.76	4.69E-02

positive regulation of RNA binding (GO:1905216)	3.76	4.69E-02
neuron cellular homeostasis (GO:0070050)	3.76	4.69E-02
histone phosphorylation (GO:0016572)	3.76	5.85E-03
protein localization to endosome (GO:0036010)	3.76	4.69E-02
regulation of microtubule nucleation (GO:0010968)	3.76	4.69E-02
BMP signaling pathway (GO:0030509)	3.76	4.69E-02
positive regulation of TORC1 signaling (GO:1904263)	3.76	4.69E-02
regulation of production of miRNAs involved in gene silencing by miRNA (GO:1903798)	3.76	4.69E-02
regulation of plasma membrane organization (GO:1903729)	3.76	4.69E-02
positive regulation of lamellipodium assembly (GO:0010592)	3.76	5.85E-03
retrograde axonal transport (GO:0008090)	3.76	4.69E-02
neuron recognition (GO:0008038)	3.76	4.69E-02
positive regulation of interleukin-10 production (GO:0032733)	3.66	1.29E-02
nucleus localization (GO:0051647)	3.66	1.29E-02
alpha-beta T cell differentiation (GO:0046632)	3.66	1.29E-02
regulation of intracellular estrogen receptor signaling pathway (GO:0033146)	3.61	3.73E-03
cytoplasmic translational initiation (GO:0002183)	3.58	3.37E-04
T cell selection (GO:0045058)	3.51	8.06E-03
mitochondrial electron transport, cytochrome c to oxygen (GO:0006123)	3.51	2.86E-02
CD4-positive, alpha-beta T cell activation (GO:0035710)	3.51	2.86E-02
regulation of RNA binding (GO:1905214)	3.51	2.86E-02
deoxyribonucleoside triphosphate metabolic process (GO:0009200)	3.51	2.86E-02
purine nucleobase biosynthetic process (GO:0009113)	3.51	2.86E-02
carbohydrate phosphorylation (GO:0046835)	3.51	2.86E-02
maintenance of cell polarity (GO:0030011)	3.51	2.86E-02
positive regulation of pattern recognition receptor signaling pathway (GO:0062208)	3.38	1.75E-02
regulation of protein deacetylation (GO:0090311)	3.19	3.84E-02
positive regulation of B cell proliferation (GO:0030890)	3.13	2.32E-02
positive regulation of axon extension (GO:0045773)	3.1	1.42E-02
negative regulation of proteasomal ubiquitin-dependent protein catabolic process (GO:0032435)	3.1	1.42E-02
nuclear pore organization (GO:0006999)	2.93	4.98E-02
regulation of heart growth (GO:0060420)	2.93	4.98E-02
nucleobase biosynthetic process (GO:0046112)	2.93	2.99E-02
positive regulation of erythrocyte differentiation (GO:0045648)	2.93	4.98E-02
immune response-regulating cell surface receptor signaling pathway involved in phagocytosis (GO:0002433)	2.93	4.98E-02
positive regulation of superoxide anion generation (GO:0032930)	2.93	4.98E-02
regulation of superoxide anion generation (GO:0032928)	2.93	4.98E-02
Fc-gamma receptor signaling pathway involved in phagocytosis (GO:0038096)	2.93	4.98E-02
negative regulation of reproductive process (GO:2000242)	2.93	4.98E-02
establishment of epithelial cell polarity (GO:0090162)	2.93	4.98E-02
establishment of spindle orientation (GO:0051294)	2.93	4.98E-02
positive regulation of axonogenesis (GO:0050772)	2.93	1.12E-02
positive regulation of TOR signaling (GO:0032008)	2.93	2.99E-02
membrane raft organization (GO:0031579)	2.93	4.98E-02

Rac protein signal transduction (GO:0016601)	2.93	4.98E-02
regulation of CD4-positive, alpha-beta T cell differentiation (GO:0043370)	2.93	4.98E-02
regulation of circadian rhythm (GO:0042752)	2.93	2.69E-03
peptide catabolic process (GO:0043171)	2.93	4.98E-02
vesicle transport along microtubule (GO:0047496)	2.93	2.99E-02
regulation of ruffle assembly (GO:1900027)	2.93	4.98E-02
T cell proliferation (GO:0042098)	2.93	4.98E-02
cell aging (GO:0007569)	2.93	6.93E-03
alpha-beta T cell activation (GO:0046631)	2.93	1.82E-02
regulation of lamellipodium assembly (GO:0010591)	2.93	1.82E-02
regulation of axonogenesis (GO:0050770)	2.83	3.38E-03
peptidyl-threonine phosphorylation (GO:0018107)	2.82	5.42E-03
apoptotic mitochondrial changes (GO:0008637)	2.81	8.72E-03
transforming growth factor beta receptor signaling pathway (GO:0007179)	2.77	2.30E-02
regulation of alpha-beta T cell differentiation (GO:0046637)	2.77	2.30E-02
response to amino acid starvation (GO:1990928)	2.74	3.78E-02
regulation of interleukin-10 production (GO:0032653)	2.74	3.78E-02
base-excision repair (GO:0006284)	2.74	3.78E-02
maintenance of protein localization in organelle (GO:0072595)	2.74	3.78E-02
positive regulation of regulated secretory pathway (GO:1903307)	2.74	3.78E-02
peptidyl-threonine modification (GO:0018210)	2.72	6.74E-03
regulation of B cell proliferation (GO:0030888)	2.7	1.08E-02
limbic system development (GO:0021761)	2.67	1.75E-02
hippocampus development (GO:0021766)	2.63	2.85E-02
positive regulation of lamellipodium organization (GO:1902745)	2.63	2.85E-02
regulation of posttranscriptional gene silencing (GO:0060147)	2.63	2.85E-02
cell fate commitment (GO:0045165)	2.63	2.85E-02
response to dsRNA (GO:0043331)	2.63	2.85E-02
regulation of axon extension (GO:0030516)	2.63	8.30E-03
regulation of gene silencing by RNA (GO:0060966)	2.63	2.85E-02
translational initiation (GO:0006413)	2.6	5.95E-04
protein modification by small protein removal (GO:0070646)	2.59	1.93E-03
nuclear membrane organization (GO:0071763)	2.58	4.68E-02
positive regulation of interleukin-2 production (GO:0032743)	2.58	4.68E-02
glycogen metabolic process (GO:0005977)	2.58	4.68E-02
cellular glucan metabolic process (GO:0006073)	2.58	4.68E-02
establishment of spindle localization (GO:0051293)	2.58	4.68E-02
glucan metabolic process (GO:0044042)	2.58	4.68E-02
positive regulation of actin filament polymerization (GO:0030838)	2.58	6.34E-03
regulation of organ growth (GO:0046620)	2.58	4.68E-02
nuclear envelope organization (GO:0006998)	2.56	2.15E-02
cell cycle G1/S phase transition (GO:0044843)	2.56	2.15E-02
histone methylation (GO:0016571)	2.56	2.15E-02
G1/S transition of mitotic cell cycle (GO:0000082)	2.56	2.15E-02
nucleus organization (GO:0006997)	2.51	8.69E-04
negative regulation of proteasomal protein catabolic process (GO:1901799)	2.51	3.48E-02
regulation of TOR signaling (GO:0032006)	2.51	3.48E-02
regulation of mitochondrial membrane permeability (GO:0046902)	2.51	3.48E-02
establishment of cell polarity (GO:0030010)	2.48	5.83E-03

regulation of extent of cell growth (GO:0061387)	2.47	1.22E-02
regulation of membrane permeability (GO:0090559)	2.46	2.60E-02
negative regulation of ubiquitin-dependent protein catabolic process (GO:2000059)	2.46	2.60E-02
positive regulation of protein polymerization (GO:0032273)	2.46	2.14E-03
regulation of intracellular steroid hormone receptor signaling pathway (GO:0033143)	2.42	1.95E-02
organelle disassembly (GO:1903008)	2.39	1.46E-02
COPII-coated vesicle budding (GO:0090114)	2.39	4.20E-02
positive regulation of plasma membrane bounded cell projection assembly (GO:0120034)	2.36	3.12E-02
erythrocyte differentiation (GO:0030218)	2.34	2.32E-02
protein methylation (GO:0006479)	2.32	1.74E-02
protein alkylation (GO:0008213)	2.32	1.74E-02
peptidyl-serine modification (GO:0018209)	2.31	2.71E-03
regulation of cell size (GO:0008361)	2.28	4.21E-03
regulation of protein targeting (GO:1903533)	2.26	2.75E-02
negative regulation of cellular protein catabolic process (GO:1903363)	2.26	2.75E-02
peptidyl-serine phosphorylation (GO:0018105)	2.24	6.52E-03
lymphocyte activation involved in immune response (GO:0002285)	2.19	3.22E-02
regulation of alpha-beta T cell activation (GO:0046634)	2.19	3.22E-02
positive regulation of developmental growth (GO:0048639)	2.19	1.79E-02
lymphocyte proliferation (GO:0046651)	2.19	4.34E-02
establishment or maintenance of cell polarity (GO:0007163)	2.16	2.86E-03
cell cycle phase transition (GO:0044770)	2.15	1.56E-02
protein autophosphorylation (GO:0046777)	2.15	1.56E-02
negative regulation of cell projection organization (GO:0031345)	2.14	2.08E-02
positive regulation of cytoskeleton organization (GO:0051495)	2.13	2.50E-03
positive regulation of angiogenesis (GO:0045766)	2.13	3.75E-02
transmembrane receptor protein serine/threonine kinase signaling pathway (GO:0007178)	2.13	3.75E-02
positive regulation of vasculature development (GO:1904018)	2.13	3.75E-02
mitotic cell cycle phase transition (GO:0044772)	2.09	2.40E-02
leukocyte chemotaxis (GO:0030595)	2.08	3.22E-02
cell activation involved in immune response (GO:0002263)	2.07	1.17E-02
DNA recombination (GO:0006310)	2.07	1.17E-02
protein deubiquitination (GO:0016579)	2.06	4.33E-02
cellular carbohydrate metabolic process (GO:0044262)	2.06	1.55E-02
macromolecule methylation (GO:0043414)	2.06	7.60E-03
response to UV (GO:0009411)	2.05	2.07E-02
leukocyte activation involved in immune response (GO:0002366)	2.03	1.77E-02
telencephalon development (GO:0021537)	2.01	2.36E-02
erythrocyte homeostasis (GO:0034101)	2.01	4.96E-02
myeloid cell homeostasis (GO:0002262)	1.97	4.21E-02
neuron projection development (GO:0031175)	1.96	5.63E-04
antigen processing and presentation (GO:0019882)	1.93	3.04E-02
plasma membrane bounded cell projection morphogenesis (GO:0120039)	1.93	7.01E-03
neuron projection morphogenesis (GO:0048812)	1.91	9.22E-03
cell projection morphogenesis (GO:0048858)	1.9	7.88E-03
histone modification (GO:0016570)	1.89	6.74E-03

regulation of plasma membrane bounded cell projection assembly (GO:0120032)	1.89	3.43E-02
protein import into nucleus (GO:0006606)	1.87	4.56E-02
regulation of cell projection assembly (GO:0060491)	1.86	3.85E-02
DNA packaging (GO:0006323)	1.86	3.85E-02
response to light stimulus (GO:0009416)	1.85	1.79E-02
regulation of protein polymerization (GO:0032271)	1.84	6.13E-03
regulation of cellular component size (GO:0032535)	1.83	1.81E-03
neuron differentiation (GO:0030182)	1.83	5.34E-04
homeostasis of number of cells (GO:0048872)	1.83	1.99E-02
cellular component morphogenesis (GO:0032989)	1.82	5.81E-03
covalent chromatin modification (GO:0016569)	1.81	1.04E-02
protein import (GO:0017038)	1.81	3.08E-02
rhythmic process (GO:0048511)	1.81	2.61E-02
forebrain development (GO:0030900)	1.81	2.61E-02
positive regulation of supramolecular fiber organization (GO:1902905)	1.8	2.22E-02
cell morphogenesis involved in neuron differentiation (GO:0048667)	1.78	3.43E-02
regulation of actin filament polymerization (GO:0030833)	1.78	2.46E-02
cell part morphogenesis (GO:0032990)	1.77	1.28E-02
regulation of microtubule-based process (GO:0032886)	1.76	3.80E-02
establishment of vesicle localization (GO:0051650)	1.76	3.80E-02
aging (GO:0007568)	1.76	1.42E-02
methylation (GO:0032259)	1.76	2.72E-02
positive regulation of protein-containing complex assembly (GO:0031334)	1.76	1.67E-02
regulation of actin filament length (GO:0030832)	1.73	2.55E-02
regulation of actin polymerization or depolymerization (GO:0008064)	1.73	2.55E-02
regulation of developmental growth (GO:0048638)	1.73	3.55E-02
axon development (GO:0061564)	1.73	4.98E-02
cellular component disassembly (GO:0022411)	1.72	1.06E-02
neuron development (GO:0048666)	1.72	3.76E-03
chemotaxis (GO:0006935)	1.71	2.38E-02
taxis (GO:0042330)	1.71	2.38E-02
positive regulation of protein kinase activity (GO:0045860)	1.7	2.61E-02
regulation of response to DNA damage stimulus (GO:2001020)	1.68	4.30E-02
DNA conformation change (GO:0071103)	1.68	2.87E-02
positive regulation of secretion by cell (GO:1903532)	1.67	3.38E-02
small GTPase mediated signal transduction (GO:0007264)	1.66	3.13E-02
peptidyl-amino acid modification (GO:0018193)	1.65	2.30E-03
regulation of actin filament organization (GO:0110053)	1.63	2.49E-02
protein modification by small protein conjugation or removal (GO:0070647)	1.62	5.54E-03
regulation of anatomical structure size (GO:0090066)	1.62	9.64E-03
positive regulation of kinase activity (GO:0033674)	1.61	2.70E-02
mitotic cell cycle process (GO:1903047)	1.6	8.93E-03
cell population proliferation (GO:0008283)	1.59	2.71E-02
cell morphogenesis involved in differentiation (GO:0000904)	1.59	4.40E-02
plasma membrane bounded cell projection organization (GO:0120036)	1.58	5.55E-03
regulation of actin cytoskeleton organization (GO:0032956)	1.58	2.31E-02
negative regulation of organelle organization (GO:0010639)	1.58	2.93E-02
generation of neurons (GO:0048699)	1.57	3.44E-03

regulation of protein-containing complex assembly (GO:0043254)	1.57	1.22E-02
cell projection organization (GO:0030030)	1.56	5.96E-03
ribonucleoprotein complex assembly (GO:0022618)	1.56	2.92E-02
positive regulation of cellular protein localization (GO:1903829)	1.54	4.00E-02
protein phosphorylation (GO:0006468)	1.52	2.42E-02
ribonucleoprotein complex subunit organization (GO:0071826)	1.52	3.64E-02
chromatin organization (GO:0006325)	1.52	1.37E-02
regulation of cytoskeleton organization (GO:0051493)	1.52	1.37E-02
positive regulation of cellular component biogenesis (GO:0044089)	1.52	2.05E-02
cell migration (GO:0016477)	1.51	1.47E-02
localization of cell (GO:0051674)	1.51	1.14E-02
cell motility (GO:0048870)	1.51	1.14E-02
positive regulation of protein transport (GO:0051222)	1.5	4.97E-02
cell morphogenesis (GO:0000902)	1.5	3.85E-02
locomotion (GO:0040011)	1.48	1.07E-02
regulation of actin filament-based process (GO:0032970)	1.47	4.40E-02
mitotic cell cycle (GO:0000278)	1.46	1.85E-02
chromosome organization (GO:0051276)	1.46	5.05E-03
neurogenesis (GO:0022008)	1.46	9.18E-03
cell cycle process (GO:0022402)	1.46	1.53E-02
positive regulation of cell differentiation (GO:0045597)	1.45	3.00E-02
cell division (GO:0051301)	1.45	3.48E-02
regulation of protein kinase activity (GO:0045859)	1.44	4.50E-02
membrane organization (GO:0061024)	1.44	9.89E-03
positive regulation of protein phosphorylation (GO:0001934)	1.43	4.67E-02
positive regulation of phosphorylation (GO:0042327)	1.42	3.84E-02
proteolysis (GO:0006508)	1.4	8.65E-03
regulation of cellular amide metabolic process (GO:0034248)	1.4	3.17E-02
positive regulation of developmental process (GO:0051094)	1.39	1.96E-02
negative regulation of cellular component organization (GO:0051129)	1.38	3.44E-02
cell cycle (GO:0007049)	1.38	9.31E-03
regulation of cell cycle (GO:0051726)	1.38	2.52E-02
apoptotic process (GO:0006915)	1.38	2.00E-02
positive regulation of organelle organization (GO:0010638)	1.37	3.95E-02
cell death (GO:0008219)	1.37	1.83E-02
positive regulation of phosphorus metabolic process (GO:0010562)	1.37	4.67E-02
positive regulation of phosphate metabolic process (GO:0045937)	1.37	4.67E-02
cellular protein modification process (GO:0006464)	1.37	1.40E-03
protein modification process (GO:0036211)	1.37	1.40E-03
intracellular signal transduction (GO:0035556)	1.34	1.50E-02
response to nitrogen compound (GO:1901698)	1.33	2.87E-02
regulation of cellular component biogenesis (GO:0044087)	1.33	3.61E-02
regulation of organelle organization (GO:0033043)	1.33	1.39E-02
movement of cell or subcellular component (GO:0006928)	1.32	3.84E-02
programmed cell death (GO:0012501)	1.32	3.84E-02
macromolecule modification (GO:0043412)	1.31	3.13E-03
cell development (GO:0048468)	1.3	4.40E-02
nervous system development (GO:0007399)	1.27	3.06E-02
protein-containing complex assembly (GO:0065003)	1.27	2.47E-02
cellular macromolecule metabolic process (GO:0044260)	1.27	7.70E-05

protein-containing complex subunit organization (GO:0043933)	1.26	1.97E-02
cellular component assembly (GO:0022607)	1.25	8.13E-03
cellular protein metabolic process (GO:0044267)	1.25	2.55E-03
protein metabolic process (GO:0019538)	1.24	1.80E-03
regulation of signal transduction (GO:0009966)	1.22	2.42E-02
cellular catabolic process (GO:0044248)	1.22	3.69E-02
cell differentiation (GO:0030154)	1.21	2.75E-02
cellular developmental process (GO:0048869)	1.21	2.53E-02
organic substance catabolic process (GO:1901575)	1.21	4.92E-02
cellular component organization (GO:0016043)	1.2	5.62E-04
catabolic process (GO:0009056)	1.19	4.51E-02
organelle organization (GO:0006996)	1.18	1.32E-02
cellular component biogenesis (GO:0044085)	1.18	2.71E-02
cellular component organization or biogenesis (GO:0071840)	1.17	1.80E-03
organonitrogen compound metabolic process (GO:1901564)	1.17	6.19E-03
multicellular organism development (GO:0007275)	1.16	4.06E-02
macromolecule metabolic process (GO:0043170)	1.15	3.30E-03
primary metabolic process (GO:0044238)	1.14	8.08E-04
nitrogen compound metabolic process (GO:0006807)	1.14	2.19E-03
organic substance metabolic process (GO:0071704)	1.11	3.03E-03
positive regulation of biological process (GO:0048518)	1.11	3.51E-02
cellular metabolic process (GO:0044237)	1.11	5.09E-03
metabolic process (GO:0008152)	1.1	3.38E-03
cellular process (GO:0009987)	1.03	4.07E-02
immune response (GO:0006955)	0.76	4.40E-02
response to biotic stimulus (GO:0009607)	0.7	1.54E-02
biological process involved in interspecies interaction between organisms (GO:0044419)	0.7	9.89E-03
response to other organism (GO:0051707)	0.65	6.24E-03
response to external biotic stimulus (GO:0043207)	0.65	6.24E-03
defense response (GO:0006952)	0.64	7.39E-03
defense response to other organism (GO:0098542)	0.58	5.32E-03
innate immune response (GO:0045087)	0.48	1.74E-03
organic acid biosynthetic process (GO:0016053)	0.47	4.57E-02
carboxylic acid biosynthetic process (GO:0046394)	0.47	4.57E-02
regulation of I-kappaB kinase/NF-kappaB signaling (GO:0043122)	0.43	4.28E-02
regulation of lipid metabolic process (GO:0019216)	0.36	3.29E-02
vascular process in circulatory system (GO:0003018)	0.21	4.75E-02
cellular response to xenobiotic stimulus (GO:0071466)	0.2	3.92E-02
humoral immune response (GO:0006959)	0.12	1.92E-03

Table S6: Enriched GO biological processes in downregulated transcripts

GO biological process complete	Fold change	raw p-value	FDR
mitochondrial translation (GO:0032543)	3.16	1.06E-05	1.65E-03
establishment of protein localization to endoplasmic reticulum (GO:0072599)	3.13	2.30E-04	2.36E-02
cytoplasmic translation (GO:0002181)	3.11	5.01E-11	2.22E-08

protein K48-linked ubiquitination (GO:0070936)	2.87	2.64E-05	3.70E-03
ribosomal large subunit biogenesis (GO:0042273)	2.76	1.34E-05	2.00E-03
protein localization to endoplasmic reticulum (GO:0070972)	2.73	5.30E-05	6.75E-03
mitochondrial gene expression (GO:0140053)	2.72	3.61E-06	6.24E-04
Golgi to plasma membrane transport (GO:0006893)	2.69	3.12E-04	3.13E-02
translation (GO:0006412)	2.66	7.12E-20	1.11E-16
peptide biosynthetic process (GO:0043043)	2.54	1.28E-18	1.53E-15
ribosomal small subunit biogenesis (GO:0042274)	2.35	3.88E-04	3.68E-02
peptide metabolic process (GO:0006518)	2.29	3.46E-18	3.83E-15
oxidative phosphorylation (GO:0006119)	2.24	6.07E-05	7.54E-03
rRNA processing (GO:0006364)	2.22	3.12E-08	7.83E-06
regulation of mRNA splicing, via spliceosome (GO:0048024)	2.18	2.14E-04	2.25E-02
amide biosynthetic process (GO:0043604)	2.16	5.53E-15	5.37E-12
rRNA metabolic process (GO:0016072)	2.15	7.37E-08	1.76E-05
ribosome biogenesis (GO:0042254)	2.14	1.65E-09	4.94E-07
post-Golgi vesicle-mediated transport (GO:0006892)	2.12	5.15E-04	4.45E-02
vesicle-mediated transport to the plasma membrane (GO:0098876)	2.08	2.04E-04	2.17E-02
protein polyubiquitination (GO:0000209)	2.03	2.02E-06	3.61E-04
protein modification by small protein removal (GO:0070646)	2.03	1.31E-04	1.51E-02
ncRNA processing (GO:0034470)	2	7.99E-10	2.70E-07
ribonucleoprotein complex biogenesis (GO:0022613)	1.95	7.17E-10	2.47E-07
protein folding (GO:0006457)	1.94	4.33E-05	5.66E-03
negative regulation of translation (GO:0017148)	1.93	1.25E-04	1.47E-02
proteolysis involved in cellular protein catabolic process (GO:0051603)	1.91	2.48E-12	1.38E-09
Golgi vesicle transport (GO:0048193)	1.9	1.22E-06	2.28E-04
endosomal transport (GO:0016197)	1.89	3.08E-05	4.27E-03
cellular protein catabolic process (GO:0044257)	1.88	3.36E-12	1.68E-09
modification-dependent protein catabolic process (GO:0019941)	1.88	1.50E-10	6.13E-08
regulation of RNA splicing (GO:0043484)	1.88	5.21E-04	4.47E-02
ncRNA metabolic process (GO:0034660)	1.86	2.90E-09	8.51E-07
modification-dependent macromolecule catabolic process (GO:0043632)	1.85	2.67E-10	1.04E-07
proteasomal protein catabolic process (GO:0010498)	1.85	4.74E-07	9.55E-05
proteasome-mediated ubiquitin-dependent protein catabolic process (GO:0043161)	1.84	2.48E-06	4.34E-04
ubiquitin-dependent protein catabolic process (GO:0006511)	1.84	1.18E-09	3.74E-07
negative regulation of cellular amide metabolic process (GO:0034249)	1.83	2.11E-04	2.23E-02
protein catabolic process (GO:0030163)	1.81	1.64E-11	7.70E-09
cellular amide metabolic process (GO:0043603)	1.81	1.04E-12	6.71E-10
regulation of protein stability (GO:0031647)	1.79	1.08E-05	1.66E-03
ribonucleoprotein complex assembly (GO:0022618)	1.79	4.43E-04	4.12E-02
cellular macromolecule catabolic process (GO:0044265)	1.78	5.32E-13	3.93E-10
ribonucleoprotein complex subunit organization (GO:0071826)	1.77	4.91E-04	4.31E-02
RNA processing (GO:0006396)	1.76	5.66E-13	4.00E-10

protein modification by small protein conjugation or removal (GO:0070647)	1.76	1.18E-12	7.32E-10
protein ubiquitination (GO:0016567)	1.72	1.61E-09	4.91E-07
protein modification by small protein conjugation (GO:0032446)	1.72	4.06E-10	1.50E-07
macromolecule catabolic process (GO:0009057)	1.71	3.00E-12	1.55E-09
gene expression (GO:0010467)	1.67	8.32E-24	2.15E-20
regulation of mRNA metabolic process (GO:1903311)	1.67	1.70E-04	1.88E-02
vesicle organization (GO:0016050)	1.63	4.81E-04	4.27E-02
RNA metabolic process (GO:0016070)	1.63	3.79E-15	3.92E-12
regulation of translation (GO:0006417)	1.62	4.26E-05	5.60E-03
cellular nitrogen compound biosynthetic process (GO:0044271)	1.62	3.06E-13	2.37E-10
histone modification (GO:0016570)	1.58	2.60E-04	2.64E-02
mRNA metabolic process (GO:0016071)	1.57	6.12E-06	1.01E-03
regulation of cellular amide metabolic process (GO:0034248)	1.56	5.79E-05	7.26E-03
covalent chromatin modification (GO:0016569)	1.56	3.81E-04	3.65E-02
posttranscriptional regulation of gene expression (GO:0010608)	1.55	4.08E-05	5.41E-03
RNA splicing (GO:0008380)	1.54	5.70E-04	4.82E-02
cellular macromolecule biosynthetic process (GO:0034645)	1.52	1.37E-10	5.74E-08
cellular protein metabolic process (GO:0044267)	1.51	2.44E-25	1.26E-21
mRNA processing (GO:0006397)	1.5	3.25E-04	3.24E-02
macromolecule biosynthetic process (GO:0009059)	1.5	2.68E-10	1.02E-07
positive regulation of cellular catabolic process (GO:0031331)	1.5	4.62E-04	4.17E-02
mitochondrion organization (GO:0007005)	1.49	5.95E-04	5.00E-02
organonitrogen compound catabolic process (GO:1901565)	1.48	2.15E-07	4.70E-05
nucleic acid metabolic process (GO:0090304)	1.45	1.32E-12	7.89E-10
cellular catabolic process (GO:0044248)	1.45	4.44E-10	1.60E-07
organonitrogen compound biosynthetic process (GO:1901566)	1.44	8.51E-08	2.00E-05
cellular nitrogen compound metabolic process (GO:0034641)	1.44	1.72E-19	2.42E-16
proteolysis (GO:0006508)	1.44	3.95E-07	8.07E-05
intracellular transport (GO:0046907)	1.42	2.92E-07	6.21E-05
chromatin organization (GO:0006325)	1.4	3.32E-04	3.28E-02
cellular macromolecule metabolic process (GO:0044260)	1.4	4.67E-24	1.45E-20
nucleobase-containing compound metabolic process (GO:0006139)	1.4	1.65E-12	9.49E-10
protein metabolic process (GO:0019538)	1.39	3.18E-19	4.12E-16
cellular protein-containing complex assembly (GO:0034622)	1.39	1.75E-04	1.92E-02
heterocycle metabolic process (GO:0046483)	1.38	2.85E-12	1.53E-09
organic substance catabolic process (GO:1901575)	1.38	1.74E-07	3.86E-05
heterocycle biosynthetic process (GO:0018130)	1.38	2.20E-04	2.28E-02
nucleobase-containing compound biosynthetic process (GO:0034654)	1.37	4.51E-04	4.12E-02
catabolic process (GO:0009056)	1.36	3.21E-08	7.93E-06
macromolecule metabolic process (GO:0043170)	1.35	4.85E-27	3.77E-23
establishment of protein localization (GO:0045184)	1.35	9.16E-06	1.47E-03
vesicle-mediated transport (GO:0016192)	1.35	8.31E-06	1.35E-03
cellular aromatic compound metabolic process (GO:0006725)	1.34	1.15E-10	4.95E-08

negative regulation of cellular protein metabolic process (GO:0032269)	1.34	2.20E-04	2.30E-02
protein transport (GO:0015031)	1.33	4.51E-05	5.84E-03
macromolecule modification (GO:0043412)	1.33	1.53E-10	6.11E-08
negative regulation of protein metabolic process (GO:0051248)	1.32	3.95E-04	3.72E-02
cellular protein modification process (GO:0006464)	1.31	7.87E-09	2.14E-06
protein modification process (GO:0036211)	1.31	7.87E-09	2.11E-06
cellular biosynthetic process (GO:0044249)	1.31	8.93E-08	2.07E-05
cellular metabolic process (GO:0044237)	1.31	1.94E-28	3.01E-24
nitrogen compound metabolic process (GO:0006807)	1.3	8.13E-23	1.58E-19
organic cyclic compound metabolic process (GO:1901360)	1.29	4.53E-09	1.28E-06
establishment of localization in cell (GO:0051649)	1.29	2.60E-05	3.68E-03
organic substance biosynthetic process (GO:1901576)	1.29	3.88E-07	8.04E-05
primary metabolic process (GO:0044238)	1.28	2.41E-23	5.36E-20
organonitrogen compound metabolic process (GO:1901564)	1.28	5.97E-14	5.16E-11
biosynthetic process (GO:0009058)	1.27	1.22E-06	2.26E-04
cellular macromolecule localization (GO:0070727)	1.26	3.87E-04	3.69E-02
cellular protein localization (GO:0034613)	1.26	4.58E-04	4.16E-02
metabolic process (GO:0008152)	1.26	2.83E-24	1.10E-20
organic substance metabolic process (GO:0071704)	1.25	2.25E-21	3.88E-18
cellular localization (GO:0051641)	1.25	1.10E-05	1.67E-03
nitrogen compound transport (GO:0071705)	1.25	3.34E-04	3.29E-02
negative regulation of nitrogen compound metabolic process (GO:0051172)	1.22	7.67E-05	9.32E-03
protein localization (GO:0008104)	1.21	4.91E-04	4.33E-02
regulation of macromolecule biosynthetic process (GO:0010556)	1.2	1.10E-06	2.08E-04
cellular component biogenesis (GO:0044085)	1.2	1.61E-04	1.81E-02
regulation of nucleobase-containing compound metabolic process (GO:0019219)	1.2	1.52E-06	2.75E-04
negative regulation of cellular metabolic process (GO:0031324)	1.2	1.74E-04	1.91E-02
organelle organization (GO:0006996)	1.2	9.72E-06	1.53E-03
regulation of cellular macromolecule biosynthetic process (GO:2000112)	1.2	2.05E-06	3.62E-04
macromolecule localization (GO:0033036)	1.19	5.41E-04	4.61E-02
regulation of RNA metabolic process (GO:0051252)	1.19	1.39E-05	2.05E-03
regulation of gene expression (GO:0010468)	1.18	5.50E-07	1.10E-04
negative regulation of macromolecule metabolic process (GO:0010605)	1.18	3.52E-04	3.44E-02
regulation of biosynthetic process (GO:0009889)	1.18	7.45E-06	1.22E-03
regulation of cellular biosynthetic process (GO:0031326)	1.18	9.28E-06	1.47E-03
regulation of primary metabolic process (GO:0080090)	1.16	3.64E-07	7.64E-05
regulation of nitrogen compound metabolic process (GO:0051171)	1.16	8.03E-07	1.54E-04
regulation of cellular metabolic process (GO:0031323)	1.16	6.46E-07	1.27E-04
regulation of macromolecule metabolic process (GO:0060255)	1.15	1.34E-06	2.44E-04
regulation of metabolic process (GO:0019222)	1.13	5.55E-06	9.37E-04
cellular component organization or biogenesis (GO:0071840)	1.11	4.46E-04	4.12E-02

response to stimulus (GO:0050896)	0.89	2.03E-05	2.95E-03
cellular response to stimulus (GO:0051716)	0.87	1.81E-05	2.65E-03
developmental process (GO:0032502)	0.84	6.65E-07	1.29E-04
multicellular organism development (GO:0007275)	0.84	1.24E-05	1.86E-03
system development (GO:0048731)	0.84	2.18E-05	3.11E-03
cellular developmental process (GO:0048869)	0.83	4.85E-05	6.23E-03
cell differentiation (GO:0030154)	0.82	3.62E-05	4.98E-03
anatomical structure development (GO:0048856)	0.82	1.37E-07	3.08E-05
anatomical structure morphogenesis (GO:0009653)	0.79	1.28E-04	1.48E-02
cell surface receptor signaling pathway (GO:0007166)	0.77	7.71E-05	9.29E-03
multicellular organismal process (GO:0032501)	0.77	5.87E-15	5.36E-12
reproductive process (GO:0022414)	0.76	4.95E-04	4.32E-02
cell communication (GO:0007154)	0.76	6.41E-12	3.11E-09
reproduction (GO:0000003)	0.76	4.46E-04	4.10E-02
neurogenesis (GO:0022008)	0.75	4.70E-04	4.20E-02
signal transduction (GO:0007165)	0.75	2.10E-11	9.61E-09
signaling (GO:0023052)	0.74	1.01E-12	6.85E-10
cell development (GO:0048468)	0.74	7.62E-05	9.32E-03
movement of cell or subcellular component (GO:0006928)	0.73	1.01E-04	1.21E-02
generation of neurons (GO:0048699)	0.73	2.33E-04	2.38E-02
cytoskeleton organization (GO:0007010)	0.72	1.95E-04	2.09E-02
negative regulation of multicellular organismal process (GO:0051241)	0.7	2.91E-04	2.94E-02
locomotion (GO:0040011)	0.69	4.05E-05	5.42E-03
nervous system process (GO:0050877)	0.66	2.04E-05	2.93E-03
system process (GO:0003008)	0.65	2.05E-08	5.23E-06
actin filament-based process (GO:0030029)	0.6	1.85E-04	2.01E-02
regulation of ion transport (GO:0043269)	0.59	5.52E-05	6.97E-03
cell junction organization (GO:0034330)	0.58	4.64E-04	4.16E-02
actin cytoskeleton organization (GO:0030036)	0.58	1.64E-04	1.84E-02
cell adhesion (GO:0007155)	0.53	1.65E-08	4.26E-06
biological adhesion (GO:0022610)	0.52	1.20E-08	3.16E-06
cell-cell adhesion (GO:0098609)	0.48	4.60E-06	7.86E-04
regulation of calcium ion transport (GO:0051924)	0.42	3.54E-04	3.42E-02
sensory perception (GO:0007600)	0.4	1.61E-09	5.00E-07
adenylate cyclase-modulating G protein-coupled receptor signaling pathway (GO:0007188)	0.37	3.54E-04	3.44E-02
G protein-coupled receptor signaling pathway (GO:0007186)	0.36	7.35E-14	6.01E-11
negative regulation of cell development (GO:0010721)	0.34	4.18E-04	3.92E-02
detection of stimulus (GO:0051606)	0.27	5.50E-09	1.53E-06
detection of abiotic stimulus (GO:0009582)	0.26	5.12E-04	4.45E-02
cilium movement (GO:0003341)	0.23	1.20E-04	1.41E-02
potassium ion transmembrane transport (GO:0071805)	0.22	6.62E-05	8.17E-03
cilium movement involved in cell motility (GO:0060294)	0.17	5.49E-04	4.66E-02
detection of chemical stimulus (GO:0009593)	0.16	2.53E-07	5.46E-05
sensory perception of smell (GO:0007608)	0.16	5.80E-06	9.68E-04
sensory perception of chemical stimulus (GO:0007606)	0.15	1.08E-07	2.46E-05
cilium-dependent cell motility (GO:0060285)	0.15	1.37E-04	1.57E-02
cilium or flagellum-dependent cell motility (GO:0001539)	0.15	1.37E-04	1.56E-02

detection of stimulus involved in sensory perception (GO:0050906)	0.1	8.46E-10	2.80E-07
cell-cell adhesion via plasma-membrane adhesion molecules (GO:0098742)	0.1	6.02E-10	2.13E-07
action potential (GO:0001508)	0.09	1.88E-04	2.03E-02
homophilic cell adhesion via plasma membrane adhesion molecules (GO:0007156)	< 0.01	3.59E-09	1.03E-06
cellular response to chemokine (GO:1990869)	< 0.01	3.72E-05	5.07E-03
response to chemokine (GO:1990868)	< 0.01	3.72E-05	5.02E-03
detection of chemical stimulus involved in sensory perception of smell (GO:0050911)	< 0.01	4.38E-08	1.06E-05
detection of chemical stimulus involved in sensory perception (GO:0050907)	< 0.01	9.05E-10	2.93E-07
chemokine-mediated signaling pathway (GO:0070098)	< 0.01	1.14E-04	1.35E-02

Table S7: Enriched GO biological processes in upregulated transcripts

**GO biological process complete**	**Fold change**	**p-value**
arginine catabolic process to proline (GO:0019493)	> 100	8.39E-03
axonemal microtubule depolymerization (GO:0060404)	> 100	8.39E-03
negative regulation of proteoglycan biosynthetic process (GO:1902729)	> 100	8.39E-03
lymphocyte chemotaxis across high endothelial venule (GO:0002518)	> 100	8.39E-03
arginine catabolic process to proline via ornithine (GO:0010121)	> 100	8.39E-03
sagittal suture morphogenesis (GO:0060367)	> 100	8.39E-03
lambdoid suture morphogenesis (GO:0060366)	> 100	8.39E-03
lateral ganglionic eminence cell proliferation (GO:0022018)	> 100	8.39E-03
subpallium cell proliferation in forebrain (GO:0022012)	> 100	8.39E-03
neural crest cell migration involved in sympathetic nervous system development (GO:1903045)	> 100	8.39E-03
negative regulation of endothelial cell chemotaxis to fibroblast growth factor (GO:2000545)	> 100	8.39E-03
negative regulation of connective tissue growth factor production (GO:0032683)	> 100	8.39E-03
fibroblast growth factor receptor apoptotic signaling pathway (GO:1902178)	> 100	8.39E-03
regulation of prostaglandin catabolic process (GO:1905828)	> 100	8.39E-03
plus-end specific microtubule depolymerization (GO:0070462)	> 100	8.39E-03
fast-twitch skeletal muscle fiber contraction (GO:0031443)	> 100	8.39E-03
negative regulation of cell chemotaxis to fibroblast growth factor (GO:1904848)	> 100	8.39E-03
endothelial cell chemotaxis to fibroblast growth factor (GO:0035768)	> 100	8.39E-03
cell chemotaxis to fibroblast growth factor (GO:0035766)	> 100	8.39E-03
triglyceride acyl-chain remodeling (GO:0036153)	> 100	8.39E-03
negative regulation of hair cycle (GO:0042636)	> 100	8.39E-03
mating plug formation (GO:0042628)	> 100	8.39E-03
xenobiotic transport across blood-nerve barrier (GO:0061772)	> 100	8.39E-03
cytoplasmic microtubule depolymerization (GO:0010938)	> 100	8.39E-03
helper T cell enhancement of adaptive immune response (GO:0035397)	> 100	8.39E-03
fumarate transport (GO:0015741)	> 100	8.39E-03

cilium disassembly (GO:0061523)	> 100	8.39E-03
endocardial cushion cell fate commitment (GO:0061445)	> 100	8.39E-03
substantia propria of cornea development (GO:1903701)	> 100	8.39E-03
endocardial cell fate commitment (GO:0060957)	> 100	8.39E-03
lateral semicircular canal development (GO:0060875)	> 100	8.39E-03
anterior semicircular canal development (GO:0060873)	> 100	8.39E-03
cellular response to butyrate (GO:1903545)	> 100	8.39E-03
response to butyrate (GO:1903544)	> 100	8.39E-03
negative regulation of lipoprotein oxidation (GO:0034443)	> 100	8.39E-03
regulation of lipoprotein oxidation (GO:0034442)	> 100	8.39E-03
seminal vesicle epithelium development (GO:0061108)	> 100	8.39E-03
seminal vesicle development (GO:0061107)	> 100	8.39E-03
arginine catabolic process to glutamate (GO:0019544)	> 100	8.39E-03
negative regulation of lipoprotein lipid oxidation (GO:0060588)	> 100	8.39E-03
regulation of lipoprotein lipid oxidation (GO:0060587)	> 100	8.39E-03
induction by virus of host cell-cell fusion (GO:0006948)	59.38	1.67E-02
frontal suture morphogenesis (GO:0060364)	59.38	1.67E-02
B-1a B cell differentiation (GO:0002337)	59.38	1.67E-02
CXCL12-activated CXCR4 signaling pathway (GO:0038160)	59.38	1.67E-02
C-X-C chemokine receptor CXCR4 signaling pathway (GO:0038159)	59.38	1.67E-02
positive regulation of macrophage migration inhibitory factor signaling pathway (GO:2000448)	59.38	1.67E-02
positive regulation of polyamine transmembrane transport (GO:1902269)	59.38	1.67E-02
positive regulation of interleukin-21 production (GO:0032745)	59.38	1.67E-02
regulation of apoptotic process involved in outflow tract morphogenesis (GO:1902256)	59.38	1.67E-02
regulation of interleukin-21 production (GO:0032665)	59.38	1.67E-02
regulation of connective tissue growth factor production (GO:0032643)	59.38	1.67E-02
positive regulation of timing of catagen (GO:0051795)	59.38	1.67E-02
mesenchymal cell migration (GO:0090497)	59.38	1.67E-02
negative regulation of bile acid biosynthetic process (GO:0070858)	59.38	1.67E-02
response to ultrasound (GO:1990478)	59.38	1.67E-02
negative regulation of smooth muscle cell-matrix adhesion (GO:2000098)	59.38	1.67E-02
positive regulation of determination of dorsal identity (GO:2000017)	59.38	1.67E-02
G1 phase (GO:0051318)	59.38	1.67E-02
larynx development (GO:0120224)	59.38	1.67E-02
larynx morphogenesis (GO:0120223)	59.38	1.67E-02
zinc ion import into synaptic vesicle (GO:0099180)	59.38	1.67E-02
trans-Golgi network membrane organization (GO:0098629)	59.38	1.67E-02
negative regulation of memory T cell differentiation (GO:0043381)	59.38	1.67E-02
cellular response to 3,3',5-triiodo-L-thyronine (GO:1905243)	59.38	1.67E-02
tricarboxylic acid transmembrane transport (GO:0035674)	59.38	1.67E-02
uterine wall breakdown (GO:0042704)	59.38	1.67E-02
mitotic G1 phase (GO:0000080)	59.38	1.67E-02
negative regulation of epithelial to mesenchymal transition involved in endocardial cushion formation (GO:1905006)	59.38	1.67E-02
negative regulation of phospholipase A2 activity (GO:1900138)	59.38	5.51E-04
zinc ion import into organelle (GO:0062111)	59.38	1.67E-02
galactose transmembrane transport (GO:0015757)	59.38	1.67E-02

negative regulation of cardiac epithelial to mesenchymal transition (GO:0062044)	59.38	1.67E-02
negative regulation of bile acid metabolic process (GO:1904252)	59.38	1.67E-02
endocardial cushion cell differentiation (GO:0061443)	59.38	1.67E-02
thymocyte migration (GO:0072679)	59.38	1.67E-02
endocardial cushion fusion (GO:0003274)	59.38	5.51E-04
agmatine biosynthetic process (GO:0097055)	59.38	1.67E-02
mammary gland specification (GO:0060594)	59.38	1.67E-02
negative regulation of serine-type peptidase activity (GO:1902572)	47.5	8.56E-04
positive regulation of epithelial to mesenchymal transition involved in endocardial cushion formation (GO:1905007)	47.5	8.56E-04
negative regulation of serine-type endopeptidase activity (GO:1900004)	47.5	8.56E-04
cell adhesion involved in heart morphogenesis (GO:0061343)	47.5	8.56E-04
atrial septum primum morphogenesis (GO:0003289)	47.5	8.56E-04
septum primum development (GO:0003284)	47.5	8.56E-04
dichotomous subdivision of terminal units involved in salivary gland branching (GO:0060666)	47.5	8.56E-04
positive regulation of integrin biosynthetic process (GO:0045726)	39.58	2.49E-02
smoothened signaling pathway involved in spinal cord motor neuron cell fate specification (GO:0021776)	39.58	2.49E-02
smoothened signaling pathway involved in ventral spinal cord interneuron specification (GO:0021775)	39.58	2.49E-02
voluntary skeletal muscle contraction (GO:0003010)	39.58	2.49E-02
positive regulation of synaptic vesicle clustering (GO:2000809)	39.58	2.49E-02
JUN phosphorylation (GO:0007258)	39.58	2.49E-02
neural fold elevation formation (GO:0021502)	39.58	2.49E-02
CD8-positive, gamma-delta intraepithelial T cell differentiation (GO:0002305)	39.58	2.49E-02
gamma-delta intraepithelial T cell differentiation (GO:0002304)	39.58	2.49E-02
putrescine biosynthetic process from ornithine (GO:0033387)	39.58	2.49E-02
negative regulation of integrin-mediated signaling pathway (GO:2001045)	39.58	2.49E-02
regulation of macrophage migration inhibitory factor signaling pathway (GO:2000446)	39.58	2.49E-02
male anatomical structure morphogenesis (GO:0090598)	39.58	2.49E-02
regulation of timing of catagen (GO:0051794)	39.58	2.49E-02
male genitalia morphogenesis (GO:0048808)	39.58	2.49E-02
negative regulation of thrombin-activated receptor signaling pathway (GO:0070495)	39.58	2.49E-02
regulation of thrombin-activated receptor signaling pathway (GO:0070494)	39.58	2.49E-02
nose morphogenesis (GO:0043585)	39.58	2.49E-02
positive regulation of mesenchymal stem cell migration (GO:1905322)	39.58	2.49E-02
regulation of mesenchymal stem cell migration (GO:1905320)	39.58	2.49E-02
response to 3,3',5-triiodo-L-thyronine (GO:1905242)	39.58	2.49E-02
allantois development (GO:1905069)	39.58	2.49E-02
regulation of epithelial to mesenchymal transition involved in endocardial cushion formation (GO:1905005)	39.58	1.23E-03
cellular magnesium ion homeostasis (GO:0010961)	39.58	2.49E-02
basal dendrite arborization (GO:0150020)	39.58	2.49E-02
basal dendrite morphogenesis (GO:0150019)	39.58	2.49E-02
basal dendrite development (GO:0150018)	39.58	2.49E-02

negative regulation of alkaline phosphatase activity (GO:0010693)	39.58	2.49E-02
chondroitin sulfate catabolic process (GO:0030207)	39.58	2.49E-02
neuroligin clustering involved in postsynaptic membrane assembly (GO:0097118)	39.58	2.49E-02
twitch skeletal muscle contraction (GO:0014721)	39.58	2.49E-02
facioacoustic ganglion development (GO:1903375)	39.58	2.49E-02
regulation of postsynaptic density protein 95 clustering (GO:1902897)	39.58	2.49E-02
regulation of serine-type peptidase activity (GO:1902571)	33.93	1.66E-03
regulation of serine-type endopeptidase activity (GO:1900003)	33.93	1.66E-03
positive regulation of proteoglycan biosynthetic process (GO:1902730)	29.69	3.31E-02
negative regulation of T-helper 2 cell differentiation (GO:0045629)	29.69	3.31E-02
tricarboxylic acid transport (GO:0006842)	29.69	3.31E-02
negative regulation of receptor recycling (GO:0001920)	29.69	3.31E-02
positive regulation of MHC class I biosynthetic process (GO:0045345)	29.69	3.31E-02
negative regulation of endothelial cell chemotaxis (GO:2001027)	29.69	3.31E-02
regulation of integrin biosynthetic process (GO:0045113)	29.69	3.31E-02
cellular response to lead ion (GO:0071284)	29.69	3.31E-02
positive regulation of hair follicle maturation (GO:0048818)	29.69	3.31E-02
regulation of smooth muscle cell-matrix adhesion (GO:2000097)	29.69	3.31E-02
negative regulation of cortisol biosynthetic process (GO:2000065)	29.69	3.31E-02
negative regulation of aldosterone biosynthetic process (GO:0032348)	29.69	3.31E-02
negative regulation of aldosterone metabolic process (GO:0032345)	29.69	3.31E-02
regulation of determination of dorsal identity (GO:2000015)	29.69	3.31E-02
negative regulation of hydrogen peroxide-mediated programmed cell death (GO:1901299)	29.69	3.31E-02
positive regulation of response to nutrient levels (GO:0032109)	29.69	3.31E-02
positive regulation of response to extracellular stimulus (GO:0032106)	29.69	3.31E-02
positive regulation of appetite (GO:0032100)	29.69	3.31E-02
ventral trunk neural crest cell migration (GO:0036486)	29.69	3.31E-02
trunk neural crest cell migration (GO:0036484)	29.69	3.31E-02
positive regulation of response to food (GO:0032097)	29.69	3.31E-02
ascending aorta morphogenesis (GO:0035910)	29.69	3.31E-02
negative regulation of aspartic-type peptidase activity (GO:1905246)	29.69	3.31E-02
acylglycerol acyl-chain remodeling (GO:0036155)	29.69	3.31E-02
protein import into peroxisome matrix, docking (GO:0016560)	29.69	3.31E-02
positive regulation of vascular wound healing (GO:0035470)	29.69	3.31E-02
negative regulation of macrophage cytokine production (GO:0010936)	29.69	3.31E-02
citrate transport (GO:0015746)	29.69	3.31E-02
alpha-ketoglutarate transport (GO:0015742)	29.69	3.31E-02
positive regulation of cardiac epithelial to mesenchymal transition (GO:0062043)	29.69	2.16E-03
synaptic vesicle uncoating (GO:0016191)	29.69	3.31E-02
trunk segmentation (GO:0035290)	29.69	3.31E-02
sympathetic neuron projection guidance (GO:0097491)	29.69	3.31E-02
sympathetic neuron projection extension (GO:0097490)	29.69	3.31E-02
positive regulation of nitric oxide mediated signal transduction (GO:0010750)	29.69	3.31E-02
negative regulation of phospholipase activity (GO:0010519)	29.69	2.16E-03
negative regulation of extracellular matrix constituent secretion (GO:0003332)	29.69	3.31E-02

smoothened signaling pathway involved in ventral spinal cord patterning (GO:0021910)	29.69	3.31E-02
establishment of blood-nerve barrier (GO:0008065)	29.69	3.31E-02
hypothalamic tangential migration using cell-axon interactions (GO:0021856)	29.69	3.31E-02
gonadotrophin-releasing hormone neuronal migration to the hypothalamus (GO:0021828)	29.69	3.31E-02
regulation of cardiac epithelial to mesenchymal transition (GO:0062042)	26.39	2.71E-03
dichotomous subdivision of an epithelial terminal unit (GO:0060600)	26.39	2.71E-03
hindgut morphogenesis (GO:0007442)	23.75	4.12E-02
negative regulation of megakaryocyte differentiation (GO:0045653)	23.75	4.12E-02
trigeminal nerve structural organization (GO:0021637)	23.75	4.12E-02
trigeminal nerve morphogenesis (GO:0021636)	23.75	4.12E-02
axonogenesis involved in innervation (GO:0060385)	23.75	4.12E-02
clathrin coat disassembly (GO:0072318)	23.75	4.12E-02
B-1 B cell differentiation (GO:0001923)	23.75	4.12E-02
negative regulation of monocyte chemotactic protein-1 production (GO:0071638)	23.75	4.12E-02
modulation by virus of host cellular process (GO:0019054)	23.75	4.12E-02
chemokine (C-X-C motif) ligand 12 signaling pathway (GO:0038146)	23.75	4.12E-02
regulation of polyamine transmembrane transport (GO:1902267)	23.75	4.12E-02
[2Fe-2S] cluster assembly (GO:0044571)	23.75	4.12E-02
negative regulation of glucocorticoid biosynthetic process (GO:0031947)	23.75	4.12E-02
negative regulation of glucocorticoid metabolic process (GO:0031944)	23.75	4.12E-02
anterograde dendritic transport of neurotransmitter receptor complex (GO:0098971)	23.75	4.12E-02
negative regulation of steroid hormone biosynthetic process (GO:0090032)	23.75	4.12E-02
positive regulation of the force of heart contraction (GO:0098735)	23.75	4.12E-02
neural crest cell migration involved in autonomic nervous system development (GO:1901166)	23.75	4.12E-02
primary amino compound biosynthetic process (GO:1901162)	23.75	4.12E-02
glucose import across plasma membrane (GO:0098708)	23.75	4.12E-02
negative regulation of lipoprotein metabolic process (GO:0050748)	23.75	4.12E-02
ascending aorta development (GO:0035905)	23.75	4.12E-02
thymocyte apoptotic process (GO:0070242)	23.75	4.12E-02
amacrine cell differentiation (GO:0035881)	23.75	4.12E-02
putrescine biosynthetic process (GO:0009446)	23.75	4.12E-02
long-chain fatty acid catabolic process (GO:0042758)	23.75	4.12E-02
L-arginine import across plasma membrane (GO:0097638)	23.75	4.12E-02
oxaloacetate transport (GO:0015729)	23.75	4.12E-02
trigeminal ganglion development (GO:0061551)	23.75	4.12E-02
neuron-glial cell signaling (GO:0150099)	23.75	4.12E-02
endocardial cell differentiation (GO:0060956)	23.75	4.12E-02
cardiac endothelial cell differentiation (GO:0003348)	23.75	4.12E-02
ductus arteriosus closure (GO:0097070)	23.75	4.12E-02
hypothalamus cell migration (GO:0021855)	23.75	4.12E-02
pathway-restricted SMAD protein phosphorylation (GO:0060389)	21.59	4.01E-03
radial glial cell differentiation (GO:0060019)	21.59	4.01E-03
forebrain dorsal/ventral pattern formation (GO:0021798)	19.79	4.93E-02
branchiomotor neuron axon guidance (GO:0021785)	19.79	4.93E-02

positive regulation of T-helper 1 cell differentiation (GO:0045627)	19.79	4.93E-02
positive regulation of cell-cell adhesion mediated by integrin (GO:0033634)	19.79	4.93E-02
optic nerve morphogenesis (GO:0021631)	19.79	4.93E-02
vesicle uncoating (GO:0072319)	19.79	4.93E-02
regulation of MHC class I biosynthetic process (GO:0045343)	19.79	4.93E-02
positive regulation of epithelial cell proliferation involved in wound healing (GO:0060054)	19.79	4.93E-02
proline biosynthetic process (GO:0006561)	19.79	4.93E-02
semaphorin-plexin signaling pathway involved in axon guidance (GO:1902287)	19.79	4.75E-03
negative regulation of T cell migration (GO:2000405)	19.79	4.93E-02
positive regulation of cardioblast differentiation (GO:0051891)	19.79	4.93E-02
peripheral nervous system axon regeneration (GO:0014012)	19.79	4.93E-02
cellular response to interleukin-15 (GO:0071350)	19.79	4.93E-02
hexose import across plasma membrane (GO:0140271)	19.79	4.93E-02
L-lysine transport (GO:1902022)	19.79	4.93E-02
regulation of macrophage colony-stimulating factor production (GO:1901256)	19.79	4.93E-02
positive regulation of vascular endothelial growth factor signaling pathway (GO:1900748)	19.79	4.93E-02
response to sucrose (GO:0009744)	19.79	4.93E-02
carbohydrate import across plasma membrane (GO:0098704)	19.79	4.93E-02
negative regulation of insulin-like growth factor receptor signaling pathway (GO:0043569)	19.79	4.93E-02
B cell chemotaxis (GO:0035754)	19.79	4.93E-02
interleukin-15-mediated signaling pathway (GO:0035723)	19.79	4.93E-02
histone dephosphorylation (GO:0016576)	19.79	4.93E-02
osteoclast development (GO:0036035)	19.79	4.93E-02
negative regulation of guanyl-nucleotide exchange factor activity (GO:1905098)	19.79	4.93E-02
L-proline biosynthetic process (GO:0055129)	19.79	4.93E-02
gamma-delta T cell differentiation (GO:0042492)	19.79	4.93E-02
cranial ganglion development (GO:0061550)	19.79	4.93E-02
hindgut development (GO:0061525)	19.79	4.93E-02
negative regulation of plasminogen activation (GO:0010757)	19.79	4.93E-02
T follicular helper cell differentiation (GO:0061470)	19.79	4.93E-02
BMP signaling pathway involved in heart development (GO:0061312)	19.79	4.93E-02
smoothened signaling pathway involved in dorsal/ventral neural tube patterning (GO:0060831)	19.79	4.93E-02
L-lysine transmembrane transport (GO:1903401)	19.79	4.93E-02
hypothalamus gonadotrophin-releasing hormone neuron development (GO:0021888)	19.79	4.93E-02
hypothalamus gonadotrophin-releasing hormone neuron differentiation (GO:0021886)	19.79	4.93E-02
L-ornithine transmembrane transport (GO:1903352)	19.79	4.93E-02
positive regulation of bicellular tight junction assembly (GO:1903348)	19.79	4.93E-02
mammary gland formation (GO:0060592)	19.79	4.93E-02
response to disaccharide (GO:0034285)	19.79	4.93E-02
semaphorin-plexin signaling pathway involved in neuron projection guidance (GO:1902285)	18.27	5.54E-03

negative regulation of lymphocyte migration (GO:2000402)	18.27	5.54E-03
regulation of phospholipase A2 activity (GO:0032429)	18.27	5.54E-03
negative regulation of mononuclear cell migration (GO:0071676)	17.81	6.90E-04
embryonic digestive tract morphogenesis (GO:0048557)	16.96	6.39E-03
type B pancreatic cell development (GO:0003323)	16.96	6.39E-03
negative regulation of type 2 immune response (GO:0002829)	16.96	6.39E-03
branching involved in salivary gland morphogenesis (GO:0060445)	15.83	7.30E-03
atrial septum morphogenesis (GO:0060413)	15.83	7.30E-03
phospholipid homeostasis (GO:0055091)	15.83	7.30E-03
negative regulation of lipase activity (GO:0060192)	14.84	8.25E-03
pharyngeal system development (GO:0060037)	14.84	1.16E-03
calcium ion import across plasma membrane (GO:0098703)	13.97	9.27E-03
salivary gland morphogenesis (GO:0007435)	13.7	1.46E-03
calcium ion import into cytosol (GO:1902656)	13.19	1.03E-02
salivary gland development (GO:0007431)	12.72	1.80E-03
regulation of monocyte differentiation (GO:0045655)	12.5	1.15E-02
positive regulation of cell adhesion mediated by integrin (GO:0033630)	12.5	1.15E-02
NLS-bearing protein import into nucleus (GO:0006607)	12.5	1.15E-02
embryonic digestive tract development (GO:0048566)	12.28	1.99E-03
glandular epithelial cell development (GO:0002068)	11.87	1.26E-02
negative regulation of cytokine production involved in inflammatory response (GO:1900016)	11.87	1.26E-02
type B pancreatic cell differentiation (GO:0003309)	11.87	1.26E-02
hyperosmotic response (GO:0006972)	11.31	1.38E-02
regulation of endothelial cell chemotaxis (GO:2001026)	11.31	1.38E-02
enteroendocrine cell differentiation (GO:0035883)	11.31	1.38E-02
positive regulation of pathway-restricted SMAD protein phosphorylation (GO:0010862)	11.05	5.19E-04
innervation (GO:0060384)	10.8	1.51E-02
regulation of insulin-like growth factor receptor signaling pathway (GO:0043567)	10.8	1.51E-02
negative regulation of steroid biosynthetic process (GO:0010894)	10.8	1.51E-02
endocardial cushion morphogenesis (GO:0003203)	10.48	3.11E-03
response to muscle stretch (GO:0035994)	10.33	1.64E-02
atrial septum development (GO:0003283)	10.33	1.64E-02
gamma-aminobutyric acid signaling pathway (GO:0007214)	9.9	1.78E-02
positive regulation of T cell migration (GO:2000406)	9.9	1.78E-02
regulation of T cell migration (GO:2000404)	9.9	3.65E-03
positive regulation of antigen receptor-mediated signaling pathway (GO:0050857)	9.9	1.78E-02
negative regulation of steroid metabolic process (GO:0045939)	9.9	1.78E-02
atrioventricular valve morphogenesis (GO:0003181)	9.9	1.78E-02
negative regulation of alpha-beta T cell differentiation (GO:0046639)	9.5	1.92E-02
semaphorin-plexin signaling pathway (GO:0071526)	9.38	4.24E-03
exocrine system development (GO:0035272)	9.38	4.24E-03
regulation of hair cycle (GO:0042634)	9.13	2.06E-02
establishment of skin barrier (GO:0061436)	9.13	2.06E-02
macrophage differentiation (GO:0030225)	9.13	2.06E-02
atrioventricular valve development (GO:0003171)	9.13	2.06E-02
negative regulation of leukocyte migration (GO:0002686)	9.13	4.55E-03

regulation of lymphocyte migration (GO:2000401)	8.96	1.12E-03
lymphocyte chemotaxis (GO:0048247)	8.91	4.88E-03
cranial nerve morphogenesis (GO:0021602)	8.8	2.21E-02
branching involved in blood vessel morphogenesis (GO:0001569)	8.8	2.21E-02
regulation of pathway-restricted SMAD protein phosphorylation (GO:0060393)	8.64	1.29E-03
regulation of water loss via skin (GO:0033561)	8.48	2.37E-02
heart trabecula morphogenesis (GO:0061384)	8.48	2.37E-02
cardiac atrium morphogenesis (GO:0003209)	8.48	2.37E-02
glandular epithelial cell differentiation (GO:0002067)	8.28	5.95E-03
endocardial cushion development (GO:0003197)	8.28	5.95E-03
negative regulation of protein processing (GO:0010955)	8.19	2.53E-02
membrane depolarization during action potential (GO:0086010)	8.19	2.53E-02
motor neuron axon guidance (GO:0008045)	8.19	2.53E-02
negative regulation of protein maturation (GO:1903318)	8.19	2.53E-02
chemokine-mediated signaling pathway (GO:0070098)	8.13	4.18E-04
negative regulation of T cell differentiation (GO:0045581)	7.92	6.74E-03
positive regulation of lymphocyte migration (GO:2000403)	7.92	2.69E-02
regulation of type 2 immune response (GO:0002828)	7.92	2.69E-02
protein localization to synapse (GO:0035418)	7.74	7.16E-03
positive regulation of calcium-mediated signaling (GO:0050850)	7.66	2.86E-02
negative regulation of smoothened signaling pathway (GO:0045879)	7.66	2.86E-02
positive regulation of ossification (GO:0045778)	7.58	7.59E-03
mesenchyme morphogenesis (GO:0072132)	7.58	7.59E-03
negative regulation of epithelial to mesenchymal transition (GO:0010719)	7.42	3.03E-02
cellular response to chemokine (GO:1990869)	7.24	7.03E-04
response to chemokine (GO:1990868)	7.24	7.03E-04
cellular defense response (GO:0006968)	7.12	8.97E-03
columnar/cuboidal epithelial cell development (GO:0002066)	6.99	3.38E-02
protein depolymerization (GO:0051261)	6.99	3.38E-02
smooth muscle cell differentiation (GO:0051145)	6.99	3.38E-02
neutrophil chemotaxis (GO:0030593)	6.99	2.76E-03
cellular biogenic amine biosynthetic process (GO:0042401)	6.99	3.38E-02
liver regeneration (GO:0097421)	6.99	3.38E-02
artery development (GO:0060840)	6.99	8.25E-04
negative regulation of lymphocyte differentiation (GO:0045620)	6.85	9.97E-03
amine biosynthetic process (GO:0009309)	6.79	3.56E-02
cardiac atrium development (GO:0003230)	6.79	3.56E-02
regulation of mononuclear cell migration (GO:0071675)	6.79	2.91E-04
ventricular septum morphogenesis (GO:0060412)	6.6	3.75E-02
monocyte chemotaxis (GO:0002548)	6.6	3.75E-02
detection of mechanical stimulus involved in sensory perception (GO:0050974)	6.6	3.75E-02
endocrine pancreas development (GO:0031018)	6.6	3.75E-02
detection of mechanical stimulus (GO:0050982)	6.6	1.10E-02
positive regulation of tyrosine phosphorylation of STAT protein (GO:0042531)	6.6	1.10E-02
granulocyte chemotaxis (GO:0071621)	6.51	3.55E-03
SMAD protein signal transduction (GO:0060395)	6.48	1.16E-02

antimicrobial humoral immune response mediated by antimicrobial peptide (GO:0061844)	6.42	3.73E-03
positive regulation of bone mineralization (GO:0030501)	6.42	3.94E-02
columnar/cuboidal epithelial cell differentiation (GO:0002065)	6.33	3.91E-03
neutrophil migration (GO:1990266)	6.33	3.91E-03
digestive tract morphogenesis (GO:0048546)	6.25	4.13E-02
calcium-dependent cell-cell adhesion via plasma membrane cell adhesion molecules (GO:0016339)	6.25	4.13E-02
endothelial cell differentiation (GO:0045446)	6.17	4.29E-03
regulation of synaptic transmission, glutamatergic (GO:0051966)	6.14	1.33E-02
positive regulation of glial cell differentiation (GO:0045687)	6.09	4.33E-02
receptor signaling pathway via JAK-STAT (GO:0007259)	6.09	4.33E-02
trabecula morphogenesis (GO:0061383)	6.09	4.33E-02
receptor localization to synapse (GO:0097120)	6.09	4.33E-02
positive regulation of mononuclear cell migration (GO:0071677)	6.04	1.40E-02
receptor signaling pathway via STAT (GO:0097696)	5.94	4.53E-02
T cell selection (GO:0045058)	5.79	4.73E-02
granulocyte migration (GO:0097530)	5.79	5.34E-03
negative regulation of alpha-beta T cell activation (GO:0046636)	5.79	4.73E-02
glial cell migration (GO:0008347)	5.79	4.73E-02
killing of cells of other organism (GO:0031640)	5.75	1.59E-02
positive regulation of transmembrane receptor protein serine/threonine kinase signaling pathway (GO:0090100)	5.71	1.99E-03
pancreas development (GO:0031016)	5.65	1.66E-02
cardiac septum morphogenesis (GO:0060411)	5.57	1.73E-02
lymphocyte migration (GO:0072676)	5.57	1.73E-02
regulation of phospholipase activity (GO:0010517)	5.4	1.87E-02
endothelial cell migration (GO:0043542)	5.32	1.95E-02
regulation of tyrosine phosphorylation of STAT protein (GO:0042509)	5.32	1.95E-02
endothelium development (GO:0003158)	5.28	7.36E-03
cellular response to interleukin-1 (GO:0071347)	5.22	7.65E-03
cell killing (GO:0001906)	5.16	7.94E-03
mononuclear cell migration (GO:0071674)	5.11	8.23E-03
response to osmotic stress (GO:0006970)	5.09	2.18E-02
gland morphogenesis (GO:0022612)	5.05	8.54E-03
BMP signaling pathway (GO:0030509)	5.02	2.26E-02
nerve development (GO:0021675)	4.95	2.35E-02
protein localization to cell junction (GO:1902414)	4.95	2.35E-02
peptidyl-tyrosine dephosphorylation (GO:0035335)	4.9	9.50E-03
neural crest cell development (GO:0014032)	4.81	2.52E-02
phagocytosis, recognition (GO:0006910)	4.75	2.60E-02
cardiac septum development (GO:0003279)	4.7	1.09E-02
endosome organization (GO:0007032)	4.69	2.69E-02
leukocyte chemotaxis (GO:0030595)	4.64	4.80E-03
mesenchymal cell development (GO:0014031)	4.51	2.97E-02
stem cell development (GO:0048864)	4.51	2.97E-02
antimicrobial humoral response (GO:0019730)	4.44	1.32E-02
myeloid leukocyte migration (GO:0097529)	4.4	1.36E-02
neural crest cell differentiation (GO:0014033)	4.29	3.36E-02
complement activation, classical pathway (GO:0006958)	4.24	3.47E-02

cellular response to BMP stimulus (GO:0071773)	4.19	3.57E-02
response to BMP (GO:0071772)	4.19	3.57E-02
transforming growth factor beta receptor signaling pathway (GO:0007179)	4.19	3.57E-02
regulation of lipase activity (GO:0060191)	4.19	3.57E-02
regulation of neural precursor cell proliferation (GO:2000177)	4.19	3.57E-02
ameboidal-type cell migration (GO:0001667)	4.17	3.46E-03
positive regulation of T cell differentiation (GO:0045582)	4.14	3.68E-02
digestive tract development (GO:0048565)	4.13	1.67E-02
synapse assembly (GO:0007416)	4.09	3.78E-02
response to interleukin-1 (GO:0070555)	4.09	1.72E-02
regulation of epithelial to mesenchymal transition (GO:0010717)	4.09	3.78E-02
regulation of organ growth (GO:0046620)	4.09	3.78E-02
epithelial cell migration (GO:0010631)	4.09	3.78E-02
humoral immune response mediated by circulating immunoglobulin (GO:0002455)	4.05	3.89E-02
stem cell differentiation (GO:0048863)	4.04	8.45E-03
epithelium migration (GO:0090132)	4	4.00E-02
negative regulation of lipid metabolic process (GO:0045833)	4	4.00E-02
cell-cell adhesion via plasma-membrane adhesion molecules (GO:0098742)	3.99	9.97E-04
morphogenesis of a branching epithelium (GO:0061138)	3.98	8.92E-03
cell chemotaxis (GO:0060326)	3.89	4.79E-03
branching morphogenesis of an epithelial tube (GO:0048754)	3.89	2.03E-02
regulation of wound healing (GO:0061041)	3.89	2.03E-02
negative regulation of extrinsic apoptotic signaling pathway (GO:2001237)	3.87	4.34E-02
digestive system development (GO:0055123)	3.86	2.08E-02
homophilic cell adhesion via plasma membrane adhesion molecules (GO:0007156)	3.81	1.07E-02
negative regulation of leukocyte cell-cell adhesion (GO:1903038)	3.8	2.19E-02
morphogenesis of a branching structure (GO:0001763)	3.76	1.13E-02
tissue migration (GO:0090130)	3.75	4.70E-02
negative regulation of leukocyte differentiation (GO:1902106)	3.71	4.82E-02
B cell receptor signaling pathway (GO:0050853)	3.67	4.94E-02
negative regulation of hemopoiesis (GO:1903707)	3.67	4.94E-02
regulation of leukocyte migration (GO:0002685)	3.67	6.31E-03
MAPK cascade (GO:0000165)	3.64	1.27E-02
liver development (GO:0001889)	3.63	2.54E-02
positive regulation of chemotaxis (GO:0050921)	3.63	2.54E-02
chemotaxis (GO:0006935)	3.58	4.28E-05
transmembrane receptor protein serine/threonine kinase signaling pathway (GO:0007178)	3.58	1.37E-02
immunoglobulin production (GO:0002377)	3.57	2.67E-02
hepaticobiliary system development (GO:0061008)	3.57	2.67E-02
taxis (GO:0042330)	3.56	4.58E-05
production of molecular mediator of immune response (GO:0002440)	3.52	2.80E-02
cellular response to transforming growth factor beta stimulus (GO:0071560)	3.52	2.80E-02
import across plasma membrane (GO:0098739)	3.49	2.86E-02
positive regulation of peptidyl-tyrosine phosphorylation (GO:0050731)	3.43	1.61E-02
lipid homeostasis (GO:0055088)	3.37	3.21E-02
negative regulation of cell-cell adhesion (GO:0022408)	3.35	1.75E-02

response to transforming growth factor beta (GO:0071559)	3.35	3.28E-02
T cell differentiation (GO:0030217)	3.35	3.28E-02
calcium-mediated signaling (GO:0019722)	3.35	3.28E-02
regulation of T cell differentiation (GO:0045580)	3.32	3.35E-02
defense response to bacterium (GO:0042742)	3.29	5.90E-03
cell recognition (GO:0008037)	3.28	1.91E-02
positive regulation of epithelial cell migration (GO:0010634)	3.25	3.57E-02
positive regulation of leukocyte differentiation (GO:1902107)	3.23	3.65E-02
positive regulation of hemopoiesis (GO:1903708)	3.23	3.65E-02
humoral immune response (GO:0006959)	3.22	6.53E-03
regulation of epithelial cell migration (GO:0010632)	3.2	1.20E-02
regulation of response to wounding (GO:1903034)	3.15	3.96E-02
mesenchymal cell differentiation (GO:0048762)	3.15	3.96E-02
epithelial cell development (GO:0002064)	3.12	2.30E-02
regulation of axonogenesis (GO:0050770)	3.1	4.12E-02
cardiac chamber development (GO:0003205)	3.1	4.12E-02
negative regulation of cell activation (GO:0050866)	3.06	2.48E-02
negative regulation of cell adhesion (GO:0007162)	3.06	8.59E-03
adaptive immune response based on somatic recombination of immune receptors built from immunoglobulin superfamily domains (GO:0002460)	3.04	2.53E-02
negative regulation of Wnt signaling pathway (GO:0030178)	3.01	4.54E-02
telencephalon development (GO:0021537)	2.98	1.63E-02
sodium ion transport (GO:0006814)	2.97	4.72E-02
muscle cell differentiation (GO:0042692)	2.94	1.72E-02
leukocyte migration (GO:0050900)	2.94	2.88E-02
cardiac muscle tissue development (GO:0048738)	2.93	4.90E-02
cytokine-mediated signaling pathway (GO:0019221)	2.91	6.69E-03
mesenchyme development (GO:0060485)	2.91	2.99E-02
epithelial tube morphogenesis (GO:0060562)	2.91	1.11E-02
regulation of chemotaxis (GO:0050920)	2.9	3.04E-02
positive regulation of cell migration (GO:0030335)	2.87	1.09E-03
adaptive immune response (GO:0002250)	2.81	2.06E-03
axon guidance (GO:0007411)	2.79	3.50E-02
neuron projection guidance (GO:0097485)	2.77	3.56E-02
kidney development (GO:0001822)	2.75	2.31E-02
positive regulation of neurogenesis (GO:0050769)	2.75	3.68E-02
positive regulation of cell motility (GO:2000147)	2.74	1.60E-03
positive regulation of cytosolic calcium ion concentration (GO:0007204)	2.71	2.46E-02
regulation of developmental growth (GO:0048638)	2.71	1.58E-02
positive regulation of MAPK cascade (GO:0043410)	2.71	4.25E-03
regulation of leukocyte differentiation (GO:1902105)	2.7	2.50E-02
positive regulation of cellular component movement (GO:0051272)	2.67	1.96E-03
renal system development (GO:0072001)	2.67	2.62E-02
positive regulation of locomotion (GO:0040017)	2.67	1.99E-03
striated muscle tissue development (GO:0014706)	2.63	2.79E-02
regulation of transmembrane receptor protein serine/threonine kinase signaling pathway (GO:0090092)	2.6	4.46E-02
morphogenesis of an epithelium (GO:0002009)	2.59	8.65E-03
mononuclear cell differentiation (GO:1903131)	2.53	3.29E-02
enzyme linked receptor protein signaling pathway (GO:0007167)	2.51	3.23E-03

positive regulation of lymphocyte activation (GO:0051251)	2.51	2.27E-02
muscle tissue development (GO:0060537)	2.5	3.44E-02
regulation of hemopoiesis (GO:1903706)	2.47	2.47E-02
vasculature development (GO:0001944)	2.45	8.21E-03
cell-cell adhesion (GO:0098609)	2.44	8.44E-03
gland development (GO:0048732)	2.44	1.80E-02
cell migration (GO:0016477)	2.44	9.51E-04
negative regulation of immune system process (GO:0002683)	2.41	1.90E-02
urogenital system development (GO:0001655)	2.39	4.12E-02
regulation of cytosolic calcium ion concentration (GO:0051480)	2.38	4.18E-02
regulation of cell migration (GO:0030334)	2.38	8.45E-04
wound healing (GO:0042060)	2.34	4.52E-02
tube morphogenesis (GO:0035239)	2.32	5.94E-03
regulation of leukocyte cell-cell adhesion (GO:1903037)	2.32	4.64E-02
blood vessel development (GO:0001568)	2.31	1.68E-02
tissue morphogenesis (GO:0048729)	2.31	1.21E-02
circulatory system development (GO:0072359)	2.29	1.79E-03
regulation of cell-cell adhesion (GO:0022407)	2.29	2.49E-02
cellular divalent inorganic cation homeostasis (GO:0072503)	2.26	2.65E-02
regulation of cell motility (GO:2000145)	2.25	1.58E-03
tube development (GO:0035295)	2.23	3.23E-03
positive regulation of leukocyte activation (GO:0002696)	2.22	3.98E-02
response to bacterium (GO:0009617)	2.21	1.18E-02
regulation of cell adhesion (GO:0030155)	2.19	6.89E-03
positive regulation of immune response (GO:0050778)	2.19	2.33E-02
positive regulation of cell activation (GO:0050867)	2.16	4.53E-02
blood vessel morphogenesis (GO:0048514)	2.16	4.53E-02
regulation of locomotion (GO:0040012)	2.16	2.41E-03
divalent inorganic cation homeostasis (GO:0072507)	2.15	3.39E-02
brain development (GO:0007420)	2.15	7.96E-03
transmembrane receptor protein tyrosine kinase signaling pathway (GO:0007169)	2.14	4.69E-02
cellular calcium ion homeostasis (GO:0006874)	2.14	4.74E-02
heart development (GO:0007507)	2.12	2.75E-02
regulation of MAPK cascade (GO:0043408)	2.12	1.58E-02
cellular metal ion homeostasis (GO:0006875)	2.11	2.84E-02
muscle structure development (GO:0061061)	2.09	3.92E-02
localization of cell (GO:0051674)	2.08	4.64E-03
cell motility (GO:0048870)	2.08	4.64E-03
regulation of cellular component movement (GO:0051270)	2.07	3.57E-03
positive regulation of immune system process (GO:0002684)	2.07	8.34E-03
cell surface receptor signaling pathway (GO:0007166)	2.06	5.81E-05
response to growth factor (GO:0070848)	2.05	4.35E-02
head development (GO:0060322)	2.03	1.25E-02
locomotion (GO:0040011)	2.03	2.68E-03
epithelium development (GO:0060429)	2.02	7.85E-03
cell adhesion (GO:0007155)	2	8.32E-03
regulation of lymphocyte activation (GO:0051249)	2	4.92E-02
biological adhesion (GO:0022610)	1.99	8.73E-03
regulation of cell activation (GO:0050865)	1.97	3.21E-02

regulation of leukocyte activation (GO:0002694)	1.91	4.82E-02
immune response (GO:0006955)	1.9	2.69E-03
protein phosphorylation (GO:0006468)	1.85	4.62E-02
chemical homeostasis (GO:0048878)	1.84	1.39E-02
defense response to other organism (GO:0098542)	1.83	2.62E-02
positive regulation of intracellular signal transduction (GO:1902533)	1.83	2.16E-02
regulation of immune system process (GO:0002682)	1.83	6.57E-03
positive regulation of signal transduction (GO:0009967)	1.81	6.05E-03
negative regulation of multicellular organismal process (GO:0051241)	1.8	2.46E-02
positive regulation of response to stimulus (GO:0048584)	1.79	1.34E-03
G protein-coupled receptor signaling pathway (GO:0007186)	1.77	3.95E-02
positive regulation of cell communication (GO:0010647)	1.77	5.09E-03
positive regulation of signaling (GO:0023056)	1.77	5.38E-03
regulation of multicellular organismal development (GO:2000026)	1.74	1.57E-02
response to external stimulus (GO:0009605)	1.72	1.80E-03
response to other organism (GO:0051707)	1.7	2.23E-02
response to external biotic stimulus (GO:0043207)	1.7	2.24E-02
defense response (GO:0006952)	1.68	2.48E-02
response to biotic stimulus (GO:0009607)	1.65	2.98E-02
tissue development (GO:0009888)	1.62	2.23E-02
movement of cell or subcellular component (GO:0006928)	1.61	3.04E-02
negative regulation of response to stimulus (GO:0048585)	1.61	2.38E-02
homeostatic process (GO:0042592)	1.55	3.65E-02
immune system process (GO:0002376)	1.52	1.98E-02
regulation of intracellular signal transduction (GO:1902531)	1.51	4.30E-02
phosphate-containing compound metabolic process (GO:0006796)	1.5	4.00E-02
anatomical structure morphogenesis (GO:0009653)	1.49	2.84E-02
phosphorus metabolic process (GO:0006793)	1.48	4.47E-02
signal transduction (GO:0007165)	1.47	1.70E-03
signaling (GO:0023052)	1.45	1.77E-03
cell communication (GO:0007154)	1.44	1.69E-03
animal organ development (GO:0048513)	1.41	2.13E-02
regulation of signal transduction (GO:0009966)	1.41	2.58E-02
regulation of response to stimulus (GO:0048583)	1.41	1.05E-02
cellular developmental process (GO:0048869)	1.4	1.63E-02
cell differentiation (GO:0030154)	1.39	2.01E-02
regulation of cell communication (GO:0010646)	1.33	4.80E-02
response to stress (GO:0006950)	1.32	4.94E-02
localization (GO:0051179)	1.3	1.85E-02
cellular response to stimulus (GO:0051716)	1.27	1.57E-02
response to stimulus (GO:0050896)	1.24	1.13E-02
regulation of cellular metabolic process (GO:0031323)	0.77	3.61E-02
regulation of gene expression (GO:0010468)	0.75	4.67E-02
organonitrogen compound metabolic process (GO:1901564)	0.74	3.73E-02
organic substance metabolic process (GO:0071704)	0.72	3.18E-03
primary metabolic process (GO:0044238)	0.7	2.89E-03
metabolic process (GO:0008152)	0.67	4.04E-04
macromolecule metabolic process (GO:0043170)	0.64	1.67E-03
regulation of RNA metabolic process (GO:0051252)	0.62	1.20E-02

regulation of nucleobase-containing compound metabolic process (GO:0019219)	0.62	8.32E-03
cellular component assembly (GO:0022607)	0.61	4.38E-02
cellular metabolic process (GO:0044237)	0.61	8.88E-05
regulation of transcription, DNA-templated (GO:0006355)	0.6	1.14E-02
regulation of nucleic acid-templated transcription (GO:1903506)	0.6	1.14E-02
regulation of RNA biosynthetic process (GO:2001141)	0.6	1.11E-02
nitrogen compound metabolic process (GO:0006807)	0.59	1.62E-04
regulation of cellular biosynthetic process (GO:0031326)	0.59	4.22E-03
cellular macromolecule metabolic process (GO:0044260)	0.59	2.40E-03
regulation of cellular macromolecule biosynthetic process (GO:2000112)	0.59	4.81E-03
regulation of macromolecule biosynthetic process (GO:0010556)	0.58	4.26E-03
regulation of biosynthetic process (GO:0009889)	0.58	3.16E-03
gene expression (GO:0010467)	0.57	3.98E-02
cellular component biogenesis (GO:0044085)	0.55	1.59E-02
catabolic process (GO:0009056)	0.53	3.00E-02
protein-containing complex subunit organization (GO:0043933)	0.47	4.04E-02
cellular catabolic process (GO:0044248)	0.46	2.20E-02
cellular nitrogen compound metabolic process (GO:0034641)	0.35	5.38E-05
organic cyclic compound metabolic process (GO:1901360)	0.34	8.33E-05
heterocycle metabolic process (GO:0046483)	0.33	1.62E-04
cellular aromatic compound metabolic process (GO:0006725)	0.32	1.12E-04
protein-containing complex assembly (GO:0065003)	0.32	1.34E-02
regulation of organelle organization (GO:0033043)	0.31	1.21E-02
regulation of cellular catabolic process (GO:0031329)	0.3	3.66E-02
cellular macromolecule catabolic process (GO:0044265)	0.3	3.62E-02
nucleobase-containing compound metabolic process (GO:0006139)	0.3	1.25E-04
protein modification by small protein conjugation or removal (GO:0070647)	0.29	3.17E-02
cellular macromolecule biosynthetic process (GO:0034645)	0.28	4.27E-03
macromolecule biosynthetic process (GO:0009059)	0.27	3.65E-03
nucleic acid metabolic process (GO:0090304)	0.19	4.15E-05
RNA metabolic process (GO:0016070)	0.18	6.98E-04
DNA metabolic process (GO:0006259)	0.17	1.96E-02
organelle assembly (GO:0070925)	0.17	1.56E-02
cellular protein-containing complex assembly (GO:0034622)	0.16	1.33E-02

Table S8: Mitochondrial proteins identified in the proteome

Gene Name	Fold Change
ATPIF1	4.018548
MT-ATP8	2.197232
ATP5J	1.10103
ATP5C1	0.879601
ATP5F1	0.915456
ATP5L	0.854469
ATP5I	0.900395
ATP5D	0.871353
ATP5J2	0.849222

ATP5B	0.810028
ATP5O	0.788785
ATP5H	0.791517
ATP5A1	0.851095
MT-CO2	1.460963
COX4I1	1.347129
COX7C	1.75101
COX6C	1.577966
COX6B1	1.120388
COX6A1	2.949049
COX5A	0.916404
COX5B	0.927444
COX7A2	0.749172
COX17	2.400447
UQCRFS1	1.154948
UQCR10	6.206716
UQCRH	0.371352
UQCRQ	2.187692
UQCRB	1.219126
UQCRC1	0.982761
UQCRC2	0.976041
CYCS	1.55574
CYC1	1.125263
SDHB	1.410843
SDHA	1.104301
NDUFA12	7.461814
NDUFS6	4.618334
NDUFA8	2.969559
NDUFA2	3.035751
NDUFA10	2.095882
NDUFV1	1.912153
NDUFB5	3.319082
NDUFS5	3.243195
NDUFA6	1.825396
NDUFB8	5.01827
NDUFB7	3.407429
NDUFS7	1.646329
NDUFB4	1.535386
NDUFB3	1.529259
NDUFA5	1.383444
NDUFB10	1.4174
NDUFS2	1.336946
NDUFS1	1.174997
NDUFA4	1.009238
NDUFA9	0.925606
NDUFB9	0.922808
NDUFA13	0.924492
NDUFB1	0.949932
NDUFS3	0.669361
NDUFV2	0.635888

NDUFS8	0.501037
ACLY	1.115909
CS	0.927465
ACOT7	1.0121
ACO2	0.904633
ACOT9	5.162997
ACO1	4.33174
IDH1	1.368543
IDH3G	1.204262
IDH3B	1.132544
IDH3A	0.95235
IDH2	0.893584
SUCLG2	1.79844
ALDH5A1	4.080714
OXCT1	1.019517
DLST	1.001079
SUCLG1	1.285626
MDH2	0.8063
MDH1	1.103093
ME2	0.993052
GLUD1	1.218425
PDHA1	1.380054
FAHD1	1.245627
DLAT	1.145313
NNT	0.987826
TOMM34	1.927577
TOMM40	1.06683
TOMM22	1.016178
TOMM70	0.857848
SAMM50	1.692605
TIMM9	3.736157
TIMM50	2.915511
TIMM44	1.584089
TIMM8B	0.216065
CPT2	4.634012
LETM1	2.028459
SLC25A10	18.84246
SLC25A1	3.362308
SLC25A22	2.353646
SLC25A13	2.197166
SLC25A19	1.86867
SLC25A12	1.469386
SLC25A11	0.984992
SLC25A3	0.923116
SLC25A6	0.883156
SLC25A5	0.915794
SLC25A4	0.821475
MRPL22	7.491335
MRPL13	6.308792
MRPL3	5.048019

MRPL21	4.77883
MRPL39	3.253796
MRPL15	2.940529
MRPL49	2.714974
MRPL1	2.628413
MRPL46	7.279318
MRPL38	2.496483
MRPL4	2.371223
GLDC	7.6
ACAD9	4.4
ACADSB	3.9
HMGCL	2.5
ALDH1B1	2.4
PPIF	2.3
BDH1	2.3
ABHD10	2.2
HIBCH	2.1
ECI1	2.1
HINT2	2
SUCLG2	1.8
PPA2	1.8
ACAA2	1.7
GSR	1.7
PDHA1	1.4
HSPE1	0.9
HSPD1	0.8
MDH2	0.8
HSPA9	0.8

Table S9: Mitochondrial transcripts identified in the transcriptome

Gene name	Fold Change
MT-TF	0.483319
TOMM5	0.695822
TIMM8B	0.729636
SARS2	0.759087
PDF	0.763535
UQCRQ	0.764501
MRPL36	0.773116
MRPL12	0.775793
NDUFB7	0.779136
FIS1	0.785406
NDUFB2	0.786153
MRPS18C	0.792595
NDUFAF4	0.793272
COX7C	0.795339
MRPL1	0.799106
ATP5ME	0.802727
ATP5F1E	0.804208

ALKBH7	0.805161
MRPS28	0.807514
COX5B	0.824178
NDUFA4	0.824832
ATP5MD	0.828656
NDUFS4	0.829138
COX7A2	0.838274
NDUFB6	0.842167
COX7B	0.843464
NDUFS3	0.845565
UQCRB	0.845616
COX17	0.847244
CYC1	0.847574
SDHAF1	0.847876
IDH3B	0.854883
ATP5F1C	0.858171
SLC25A11	0.862285
SLC25A25	0.862698
SDHAF4	0.863386
ATP5IF1	0.865271
ATP5PO	0.865923
TIMM50	0.866348
COX14	0.869231
NDUFS7	0.870232
TOMM7	0.872015
NDUFA2	0.874745
TIMM13	0.885936
ATP5MC1	0.887943
NDUFAF3	0.888937
TOMM40	0.889823
SLC25A6	0.890309
NDUFV1	0.894996
COX4I2	0.753953
SLC25A41	0.756287
SLC25A47	0.796938
SLC25A10	0.84764
SLC25A29	0.850192
NDUFAF8	0.85633
SDHAF3	0.861407
NDUFV2	0.86268
NDUFA1	0.863973
COX6A1	0.864799
UQCRHL	0.873007
UQCRH	0.875275
COX20	0.894291
NDUFA11	0.895853
SLC25A42	0.896236
TIMM23B	0.897412
UQCR11	0.899887
ATP5F1D	0.901896

ATP5MC2	0.907452
COX6B1	0.920089
NDUFB9	0.921986
UQCRC1	0.928171
DLST	0.943618
NDUFS5	0.900311
TIMM10	0.903133
NDUFAF6	0.906191
IDH2	0.907576
NDUFB1	0.908102
MDH1	0.908842
MT-ND1	0.909513
SLC25A17	0.910043
ATP5MG	0.910078
SLC25A32	0.91205
COX6C	0.912577
NDUFB3	0.913949
NDUFA12	0.914104
SDHAF2	0.914988
ATP5S	0.917114
MT-ND3	0.917555
NDUFA6	0.921237
SLC25A28	0.922533
COX4I1	0.924381
NDUFAF5	0.927269
TIMM44	0.928058
UQCR10	0.928763
TIMM17B	0.929581
ATP5PB	0.929752
FH	0.930032
NDUFAF2	0.932033
ALKBH2	0.932771
TIMM29	0.93287
TOMM20	0.934633
NDUFS8	0.934937
ALKBH4	0.936381
UQCRFS1	0.93658
SLC25A39	0.937739
NDUFAF7	0.938311
ATP5PF	0.938513
SLC25A35	0.938589
COX5A	0.939493
SLC25A26	0.940685
SLC25A22	0.940793
MDH2	0.940943
SLC25A37	0.942612
COX8A	0.94297
SLC25A13	0.943048
ATP5F1B	0.943429
NDUFAB1	0.943441

SDHB	0.944961
TIMM23	0.946324
ATP5MC3	0.947152
NDUFS2	0.947567
NDUFA5	0.948533
MT-ND2	0.949129
ATP5PD	0.949266
COX19	0.950544
IDH3G	0.950926
SLC25A14	0.951504
NDUFA8	0.952186
TIMM22	0.952663
SAMM50	0.953099
MT-CYB	0.953403
ATP5MF	0.95472
SLC25A33	0.957183
SLC25A45	0.958617
NDUFB10	0.958693
TIMM10B	0.959342
SDHD	0.961767
IDH3A	0.962965
SLC25A1	0.963559
TOMM22	0.965633
TIMM8A	0.965978
NDUFB8	0.96682
UQCRC2	0.966893
COX16	0.967562
NDUFA13	0.967598
SLC25A15	0.970712
SLC25A19	0.974696
SLC25A4	0.978543
NDUFB11	0.980417
TOMM34	0.980557
COX18	0.982976
SLC25A3	0.983026
NDUFB4	0.984732
NDUFA9	0.985233
SLC25A12	0.988486
NDUFS6	0.989726
ALKBH6	0.990018
SLC25A46	0.994967
COX10	0.99525
NDUFB5	0.995801
TIMM17A	0.998263
ALKBH3	0.999385
CS	1.003083
NDUFA3	1.006012
SDHA	1.006262
SLC25A20	1.008581
SLC25A5	1.009059

TIMMDC1	1.010464
COX11	1.010713
SLC25A16	1.013231
NDUFA10	1.015352
ACO1	1.016549
NDUFC2	1.017404
MT-ND4	1.017864
ATP5F1A	1.023415
NDUFS1	1.025747
SDHC	1.029275
COX6B2	1.031563
SLC25A38	1.035799
SLC25A36	1.036067
CSKMT	1.042581
MT-ATP6	1.045895
NDUFA7	1.051238
COX15	1.052307
TIMM21	1.056458
MDH1B	1.057118
TIMM9	1.058648
NDUFV3	1.06676
MT-ND5	1.072956
SLC25A51	1.074769
TOMM70	1.075039
NDUFAF1	1.078611
SLC25A23	1.079845
MT-ND4L	1.080345
SLC25A44	1.090005
MT-ND6	1.094263
SLC25A40	1.097148
NDUFC1	1.102
SLC25A43	1.12793
SLC25A53	1.133185
SLC25A30	1.133792
IDH1	1.144834
MT-ATP8	1.171704
COX7A1	1.209119
SLC25A18	1.264148
SUGCT	1.267714
SLC25A34	1.283628
SLC25A24	1.991716
